# Comparative chloroplast genomes and phylogenetic analysis of the *Phlegmariurus* (Lycopodiaceae) from China and neighboring regions

**DOI:** 10.3389/fpls.2025.1543431

**Published:** 2025-07-08

**Authors:** Ruichen Xiang, Jiayu Hu, Javzandolgor Chuluunbat, Fei Wu, Bo Qin, Xianchun Zhang, Rihong Jiang

**Affiliations:** ^1^ State Key Laboratory of Plant Diversity and Specialty Crops, Institute of Botany, Chinese Academy of Sciences, Beijing, China; ^2^ China National Botanical Garden, Beijing, China; ^3^ College of Life Sciences, University of Chinese Academy of Sciences, Beijing, China; ^4^ Key Laboratory of Southern Subtropical Plant Diversity, Fairy Lake Botanical Garden, Shenzhen, China; ^5^ Laboratory of Plant Systematics and Phylogenetic, Botanic Garden and Research Institute, Mongolian Academy of Sciences, Ulaanbaatar, Mongolia; ^6^ Beijing Floriculture Engineering Technology Research Centre, Beijing, China; ^7^ Guangxi Key Laboratory of Special Non-wood Forest Cultivation and Utilization, Guangxi Engineering and Technology Research Center for Woody Spices, Guangxi Laboratory of Forestry, Guangxi Forestry Research Institute, Nanning, China

**Keywords:** lycophytes, *Phlegmariurus*, chloroplast genome characters, phylogeny, infrageneric classification

## Abstract

The lycophyte genus *Phlegmariurus* (Herter) Holub (Huperzioideae, Lycopodiaceae) is ecologically and pharmaceutically significant, notably as a natural source of Huperzine A—a promising therapeutic candidate for Alzheimer’s disease. Despite its medicinal potential, taxonomic ambiguities on species delimitation and infrageneric classification have impeded conservation and sustainable utilization efforts. Here, we assembled 40 *Phlegmariurus* complete chloroplast genomes, including all taxa from China, most of which were reported for the first time. Our results revealed the conserved quadripartite architectures and little variation in genome size and GC content in the genus. Comparative analyses on genome sequences identified seven hypervariable loci as prospective DNA barcodes for species discrimination. The phylogenetic toopologies reconstructed from nuclear ribosomal DNA and chloroplast genome data consistently resolved four monophyletic clades, further validated by SNP-based discriminant analysis of principal components. They are well corresponding to the four sections’ classification on Chinese taxa (sect. *Squarrosurus*, sect. *Phlegmariurus*, sect. *Fargesiani*, sect. *Hamiltoniani*). Notably, nuclear and chloroplast data congruently yielded a sister relationship between sect. *Squarrosurus* and sect. *Phlegmariurus*. However, the phylogenetic positions of sect. *Fargesiani* and sect. *Hamiltoniani* conflicted across different datasets. The diversification of the Chinese *Phlegmariurus* was traced back to the Oligocene (ca. 26.04 Ma). The comprehensive genetic resources generated herein provide a foundation for future research on species identification, population genomics and genetic diversity preservation in this medicinally significant vital genus.

## 1 Introduction

*Phlegmariurus* (Huperzioideae, Lycopodiaceae) is a lycophyte genus that is mainly epiphytic or lithophytic with approximately 250 species and renowned for its medicinal properties (PPGI, 2016). There are 21 species in China, some of which have long been used as traditional Chinese herbal medicines (Jiang et al., 2023a; Xu et al., 2019). The processed whole plant is utilized for a variety of therapeutic purposes, including pain relief, detoxification, treatment of injuries and contusions, alleviation of joint swelling and pain, and as a treatment for poliomyelitis (Xu et al., 2019; Yang, 1988a). Moreover, *Phlegmariurus* was regarded as significant source of Huperzine-A (HupA; also known as fordine), which was first isolated from *Phlegmariurus fordii* (Yang, 1988b; Xu et al., 1985). HupA is a potent, reversible, and selective acetylcholinesterase inhibitor (AChEI) and has demonstrated efficacy in the treatment of Alzheimer’s disease (AD) (Ferreira et al., 2016; Bai, 2007; Little et al., 2008; Zhou et al., 2001; Wu et al., 2011). It has been successfully extracted from 16 *Phlegmariurus* species, and the *Phlegmariurus* species produce higher HupA concentrations than *Huperzia* species (Ma et al., 2005; Ma and Gang, 2008). Study on *P. tetrastichus* identified the key genes involved in HupA synthesis pathway offering insights into its biosynthetic mechanism (Nett et al., 2021), which highlighted the significance in the advancement of contemporary pharmaceuticals research (Ma and Gang, 2008). In addition, the epiphytic *Phlegmariurus* species, found on tree trunks or rocks in forests, are popular ornamental plants due to their elegant foliage and pendant growth form (Jiang and Zhang, 2022). However, intensified harvesting for medicinal and horticultural purposes has led to severe natural resource declines. Now, all Chinese *Phlegmariurus* species are listed in the Grade II Category of the List of National Key Protected Wild Plants of China (State Forestry and Grassland Administration and the Ministry of Agriculture and Rural Affairs, P. R. China, 2021).

Clarifying the phylogenetic relationships of *Phlegmariurus* is critical for developing targeted conservation strategies. The available global phylogenetic analyses congruently identified two major clades corresponding well with the Neotropical and the Paleotropical lineages respectively by few chloroplast DNA fragments (Field et al., 2016; Wikström and Kenrick, 1997, 2000, 2001; Wikström et al., 1999; Testo et al., 2018a; Bauret et al., 2018). Most mainly focused on the Neotropical species (Field et al., 2016; Wikström and Kenrick, 1997, 2000, 2001; Wikström et al., 1999; Testo et al., 2018a). Most studies have focused on the Neotropical species (Field et al., 2016; Wikström and Kenrick, 1997, 2000, 2001; Wikström et al., 1999; Testo et al., 2018a), while the phylogeny of the Paleotropical Phlegmariurus, which contains about 100 species, has only been studied by Bauret et al. (2018), with sampling concentrated in the Western Indian Ocean region. The monophyly of the Malagasy species was recognized with strong support, however, the other Paleotropical congeners exhibited insufficient resolution. For the Chinese *Phlegmariurus*, several infrageneric classifications were proposed based on morphological characters (Ching, 1982; Yang, 1990; Zhang and Kung, 1999; 2000). Recently, four sections’ classification was presented by combining molecular and morphological data: sect. *Fargesiani*, sect. *Hamiltoniani*, sect. *Phlegmariurus*, and sect. *Squarrosurus* (Jiang et al., 2023a). The above classifications were summarized in **Supplementary Table S1**.

Currently, chloroplast genome data are widely used in phylogenetic analyses and species identifications across seed plants to cryptogams (Dobrogojski et al., 2020; Fu et al., 2022; Jiang et al., 2023b; Ma et al., 2024; Wei and Zhang, 2020; 2022; Yang et al., 2022; Zhang et al., 2020; 2022). Although chloroplast genomes of *Phlegmariurus* have been reported for several species (Tang et al., 2020; Luo et al., 2019), a comprehensive study or comparative analysis within the genus is still lacking. Nuclear ribosome DNA (nrDNA) is frequently employed to reconstruct phylogenetic relationships and resolve taxonomic ambiguities among species. These sequences are highly repetitive, with thousands of tandemly arranged copies across multiple chromosomal loci. Analyzing both nrDNA and chloroplast genomes successfully reveals intricate patterns of genetic diversity and phylogenetic relationships especially among closely related species (Wei et al., 2017; Wei and Zhang, 2020; Wei et al., 2021; Wei and Zhang, 2022; Zhang et al., 2020; Acosta and Premoli, 2010).

In this study, we undertook an intense sampling from China and neighboring regions, including 40 individuals encompassing all recognized Chinese *Phlegmariurus* species. Our objectives are (1) to investigate the overall structure and sequence characteristics of the *Phlegmariurus* chloroplast genomes from China and neighboring regions; (2) to identify and analyze the rapidly evolving genome regions, including divergent hotspots and SSRs that may serve as valuable tools for future species identification, phylogenetic, and phylogeographic studies; and (3) to explore the phylogenetic outcomes derived from both chloroplast genome and nr DNA, further to reassess previous sectional classifications. This study will inform pharmaceutical resource identification and cultivation, further shed insight into conservation efforts.

## 2 Materials and methods

### 2.1 Taxon sampling, DNA extraction and sequencing

Forty individuals were sampled from field collections or herbarium specimens, along with two supplementary samples retrieved from NCBI, collectively representing 22 recognized *Phlegmariurus* species distributed in China, Myanmar, Vietnam, Thailand, and Japan. This sampling encompassed all 21 *Phlegmariurus* species currently recognized in China. Field samplings were permitted by natural reserves in Xizang, Zhejiang, Sichuan, Yunnan, Hubei, Guangdong, Guangxi, Fujian, and Hainan provinces in China. The vouchers were identified by Prof. Xian-Chun Zhang and Dr. Ri-Hong Jiang, and deposited in the Herbarium of Institute of Botany, Chinese Academy of Sciences (PE). The voucher information is presented in **Table 1**. Multiple individuals were included where feasible to account for potential intraspecific variation and site variation. The chloroplast genomes of *P. phlegmaria* and *P. carinatus* from NCBI (MT78212 and ON773236) were included in our dataset. Total genomic DNA was extracted from silica-dried leaf tissues using the Plant Genomic DNA Kit (Tiangen Biotech CO. LTD., Beijing, China). The quality and concentrations of the DNA were assessed using agarose gel electrophoresis and a Qubit 3.0 Fluorometer (Life Technologies). Paired-end libraries (150 bp read length, 350 bp insert size) were prepared and subsequently sequenced on the Illumina NovaSeq 6000 platform (Novogene Co., Ltd., Beijing, China), yielding approximately 6–8 Gb raw reads per sample. The Illumina sequencing data were deposited into the NCBI Sequence Read Archive (SRA) under the BioProject accession number: PRJNA1241535.

**Table 1:** Overview of new generated voucher information of *Phlegmariurus* in this study.

Species	Voucher	Locality	Collector	Herbarium
*P. sieboldii*	01310980	Japan	Miyoshi	PE
*P. sieboldii*	01563712	Japan	Tagawa	PE
*P. yunfengii*	huang-11	Yunnan, China	Y.-F. Huang	PE
*P. yunnanensis*	17650	Yunnan, China	W.-M. Zhu	PE
*P. fargesii*	91135	Guangxi, China	J.-X. Zhong	PE
*P. cancellatus*	P01909	Xizang, China	B.-S. Li	PE
*P. cancellatus*	13023	Xizang, China	H. Wang	PE
*P. cancellatus*	1441	Xizang, China	Qingzang team	PE
*P. pulcherrimus*	5283	Xizang, China	X.-C. Zhang	PE
*P. pulcherrimus*	683	Xizang, China	Y.-S. Chen	PE
*P. ovatifolius*	2210	Myanmar	X.-H. Jin	PE
*P. hamiltonii*	13034	Yunnan, China	R.-H. Jiang	PE
*P. hamiltonii*	13212	Yunnan, China	R.-H. Jiang	PE
*P. cryptomerinus*	3074	Fujian, China	Longxi-Expedition	PE
*P. mingcheensis*	9551	Zhejiang, China	X.-C. Zhang	PE
*P. petiolatus*	WXP324	Yunnan, China	X.-P. Wei	PE
*P. petiolatus*	CBL010	Guangdong, China	X.-C. Zhang	PE
*P. petiolatus*	80899	Yunnan, China	Y.-M. Shui	PE
*P. petiolatus*	13053	Guangxi, China	R.-H. Jiang	PE
*P. petiolatus*	13195	Guangxi, China	R.-H. Jiang	PE
*P. petiolatus*	13042	Guangxi, China	R.-H. Jiang	PE
*P. obovalifolius*	1264	Vietnam	S. G. WU	PE
*P. fordii*	13058	Hubei, China	J.-X. Zhong	PE
*P. fordii*	2896	Tibet, China	X.-C. Zhang	PE
*P. fordii*	20170406	Fujian, China	Q. He	PE
*P. cunninghamioides*	9640	Guangxi, China	X.-C. Zhang	PE
*P. cunninghamioides*	13196	Guangxi, China	R.-H. Jiang	PE
*P. shingianus*	3325	Guangxi, China	R.-H. Jiang	PE
*P. henryi*	13220	Guangxi, China	Shiwandashan- Expedition	PE
*P. henryi*	D1923	Guangxi, China	L. Wu	PE
*P. henryi*	3046	Guangxi, China	Shiwandashan- Expedition	PE
*P. guangdongensi*	1936	Hainan, China	Hainan-Expedition	PE
*P. guangdongensi*	D66	Hainan, China	S.-Y. Dong	PE
*P. guangdongensi*	D574	Hainan, China	S.-Y. Dong	PE
*P. subulifolius*	9084	Yunnan, China	Tibetan-Expedition	PE
*P. subulifolius*	13057	Yunnan, China	E.-F Huang	PE
*P. squarrosus*	13029	Hainan, China	R.-H. Jiang	PE
*P. carinatus*	13044	Guangxi, China	R.-H. Jiang	PE
*P. salvinioides*	13227	Thailand	E.-F Huang	PE
*P. phlegmaria*	1330	Hainan, China	X.-C. Zhang	PE

### 2.2 Chloroplast genome assembling, annotation and nrDNA extraction

Quality assessment of raw reads was performed using FastQC (Wingett and Andrews, 2018) to ensure the low-quality reads being removed. The chloroplast genomes of 40 *Phlegmariurus* individuals were assembled using GetOrganelle pipeline (http://github.com/Kinggerm/GetOrganelle) (Jin et al., 2020), using *H. serrata* (NC_033874), *H. lucidula* (NC_006861), *P. phlegmaria* and *P. carinatus* (MT78212 and ON773236) as references (Tang et al., 2020; Wolf et al., 2005; Guo et al., 2016). Assembly parameters were configured according to the online manual. The assembled data were annotated using GENEIOUS v. 11.1.4 (Kearse et al., 2012) and chloroplast genome map was drawn using OGDRAW (Greiner et al., 2019). To validate assembly accuracy, consensus sequences from GetOrganelle were remapped to raw Illumina reads in GENEIOUS v. 11.1.4. The nrDNA were extended and assembled from Illumina reads using GetOrganelle. The nrDNA region analyzed in our study encompasses the complete internal transcribed spacer (ITS) regions (ITS1 and ITS2) and the nuclear ribosomal RNA genes (18S, 5.8S, and 26S), which were extracted with the k-mer size used in SPAdes set as 35, 85, and 115.

Gene statistics (number, length, and GC content) were calculated using GENEIOUS v. 11.1.4. The boundaries of large single copy (LSC), small single copy (SSC) and inverted repeat regions (IRs) of chloroplast genomes were determined using the online program IRscope (Amiryousefi et al., 2018), and the protein-coding genes were extracted in GENEIOUS v. 11.1.4. All newly annotated *Phlegmariurus* nrDNA sequences and chloroplast genomes were deposited into the NCBI GenBank database (accession numbers: PP944823–PP944846; PP419991–PP420030).

### 2.3 Repeat analyses

Tandem repeats (≥ 10 bp) were identified using the online program Tandem Repeats Finder (http://tandem.bu.edu/trf/trf.html) (Benson, 1999). Simple sequence repeats (SSRs) were calculated in MISA-web (http://webblast.ipk-gatersleben.de/misa/) (Beier et al., 2017). The minimum number of repetitions was set to 10, 5, 4, 3, 3, and 3 for mononucleotide, dinucleotides, trinucleotides, tetranucleotides, pentanucleotides and hexanucleotide repeats, respectively. The size and position of repeat sequences were assessed by REPuter (Kurtz et al., 2001), including inverted (palindromic), direct (forward), reverse, and complement repeats. Short dispersed repeats (SDRs) were also detected using REPuter. The following constraint sets for repeat identification were used: (1) 90% greater sequence identity; (2) hamming distance equal to 3; and (3) a minimum repeat size of 30 bp. The Maximum length of sequence between two SSRs to register as compound SSR was set to 0.

### 2.4 Adaptive evolution and codon usage analysis

To investigate the selective pressures acting on protein-coding genes, the site-specific models implemented in the codeml package of PAMLX (Yang and Bielawski, 2000; Xu and Yang, 2013; Yang et al., 2000; 2005; Goldman and Yang, 1994) were employed to estimate the nonsynonymous (dN) and synonymous (dS) substitution rates, as well as their ratio (ω = dN/dS). First, the unique functional protein-coding sequences for each gene were extracted and aligned using GENEIOUS and the MEGA11 MUSCLE (Codons) alignment tool. Subsequently, maximum likelihood phylogenetic trees were constructed based on the complete chloroplast genomes using RAxML v7.2.8 (Kozlov et al., 2019).

The site-specific model in PAML was utilized to allow the ω to vary among sites while maintaining a fixed ω across all branches. This approach enabled the testing for site-specific evolution within the gene phylogeny (Yang et al., 2005). Two likelihood ratio tests were performed to detect positively selected sites: Model 1 (neutral) vs. Model 2 (positive selection) and Model 7 (beta) vs. Model 8 (beta and ω). These tests compared different site-specific models to identify sites under positive selection (Yang et al., 2000; 2005; Fan et al., 2018).

M1 categorized sites into two classes with ω < 1 and ω = 1, representing negative selection and neutral evolution, while M2 introduced a third class with ω > 1 to account for positive selection. M7 and M8 described the distribution of ω using a beta function, with M7 restricting ω to the range (0, 1) and M8 allowing for additional site classes with ω > 1 to capture positive selection. Sites identified as candidates for positive selection were further evaluated based on significant posterior probability support [*: p(ω > 1) ≥ 0.95; **: p(ω > 1) ≥ 0.99] using both Naive Empirical Bayes (NEB) analysis and Bayes Empirical Bayes approach (Yang et al., 2005) identified by M2 and M8.

Codon usage analysis for protein-coding genes were measured by the relative synonymous codon usage (RSCU) values, which reflect the usage bias of synonymous codons (Behura and Severson, 2012). The PCGs were extracted using a Perl script, and the RSCU values were calculated using MEGA v11.0.11 (Tamura et al., 2021). The codon usage bias was visualized using an R script.

### 2.5 Comparative analyses of chloroplast genome

To identifying hypervariable regions in the *Phlegmariurus* chloroplast genome for future genetic population and species identification studies, a sliding window analysis was conducted in DnaSP v.6.12.03 (http://www.ub.edu/dnasp/) (Rozas et al., 2017) Nucleotide diversity (Pi) was calculated across all protein-coding and noncoding (intron and intergenic spacer) regions. The analysis was conducted on an alignment of 42 *Phlegmariurus* chloroplast genomes which were aligned in MAFFT v.7 (Katoh and Standley, 2013) and manually adjusted with GENEIOUS v. 11.1.4. The sliding window width was set to 600 bp and the step size was set to 200 bp. Regions exhibiting both aligned lengths exceeding 600 bp and nucleotide diversity values (Pi) greater than 0.04 were selected as candidate markers for species delimitation. Percentage and the number of variable sites across the total 87 PCGs of the *Phlegmariurus* chloroplast genomes was quantified using MEGA v11.0.11 (Tamura et al., 2021). Each PCG was extracted from the 42 *Phlegmariurus* chloroplast genomes and aligned in MAFFT v.7 (Katoh and Standley, 2013).

Discriminant Analysis of Principal Components (DAPC) was implemented to delineate genetic clusters and resolve complex population structures among the samples based on the single nucleotide polymorphorphisms (SNPs) data (Jombart et al., 2010). This analysis retained the first two principal components, which explained the highest variance in the data, for the subsequent genetic structure analysis. SNPs were extracted from chloroplast genome alignments using the package *adegent* (https://github.com/thibautjombart/adegenet) (V. 2.1.10) in R (Jombart, 2008; Jombart and Ahmed, 2011).

### 2.6 Phylogenetic analyses

Phylogenetic analyses were conducted based on three datasets: (1) the complete chloroplast genome sequence dataset (168,430 bp) of 44 individuals representing 24 species, (including two outgroup), (2) the concatenated 87 protein-coding gene sequence dataset (67,030 bp) of 42 individuals representing 22 species, and (3) the complete nrDNA dataset (6,498 bp) of 24 individuals, representing 19 species. Each dataset was aligned using MAFFT v.7 and then manually checked and concatenated in GENEIOUS v. 11.1.4. *Lycopodium clavatum* (NC_040994) and *Huperzia serrata* (NC_033874) were selected as outgroups. *Huperzia* was chosen as an outgroup due to its close relationship with *Phlegmariurus* in the same subfamily Huperzioideae. *Lycopodium clavatum*, a member of the subfamily Lycopodioideae, which is closely related to Huperzioideae, was selected to provide a more distantly related reference point for rooting the phylogenetic tree (PPGI, 2016).

To identify the optimal nucleotide substitution model for each dataset, ModelTest-NG v.3 (Diego et al., 2020) were used under the corrected Akaike Information Criterion (AICc) and the default option in ModelTest-NG was applied to find the best-fit model respectively. The model of nrDNA nucleotide substitutions for the Maximum Likelihood (ML) and Bayesian inferences (BI) analyses were GTR+G4. The substitution models of both chloroplast genome datasets were GTR+I+G4. All ML analyses were performed in RAxML-NG v1.0.1 (Kozlov et al., 2019) under each model, and 1,000 rapid bootstrap replicates were run to evaluate the support values (BS) for each node. BI analyses were conducted in MrBayes V.3.2.6 (Ronquist and Huelsenbeck, 2003) based on the same datasets as above. The substitution model of MrBayes was calculated in ModelFinder (Kalyaanamoorthy et al., 2017). Two MCMC runs were performed simultaneously with five million generations and four chains, sampling every 5,000 generations, and discarding 25% as burn-in. The consensus tree was constructed from the remains to estimate posterior probabilities (PP). The ML support values, and posterior probabilities were checked in Figtree V.1.4.4. (http://tree.bio.ed.ac.uk/software/figtree/).

### 2.7 Estimation of divergence time

Divergence times among lineages were estimated using a Birth Death Model with optimized relaxed clock in BEAST v2.7.6 (Drummond and Rambaut, 2007). Lycophytes include three families: Lycopodiaceae, Isoetaceae, and Selaginellaceae. Due to the absence of reliable fossils, we employed secondary calibration from prior studies (Bauret et al., 2018; Testo et al., 2018b) and fossil calibration for the divergence time of Isoetaceae and Selaginellaceae within lycophytes (Grierson and Banks, 1963). The analysis included *Huperzia serrata*, *Phylloglossum drummondii*, *Isoetes malinverniana*, *I. japonica*, *Selaginella tamariscina*, and *S. moellendorffii* for molecular dating. The input sequences was constructed by concatenating chloroplast protein-coding genes from 28 aligned species, with fossil and secondary calibration points incorporated as temporal constraints: (1) the crown node of *Phlegmariurus* and *Huperzia* was assigned a normal prior distribution (mean = 79.1 Ma, σ = 18.95; 95% HPD: 46.2–122 Ma) based on secondary calibration from Bauret et al. (2018); (2) the stem node of homosporous and heterosporous lycophytes was constrained with a normal prior (mean = 404 Ma, σ = 5; 95% HPD: 394–414 Ma) following Testo et al. (2018b); (3) the crown node of *Isoetes* and *Selaginella* was calibrated using a fossil-derived normal prior (mean = 382 Ma, σ = 6; 95% HPD: 372–392 Ma) based on macrofossil evidence from Grierson and Banks (1963). The nucleotide substitution model was set to GTR based on the results of Modelfinder (Kalyaanamoorthy et al., 2017). The analyses were run for 200,000,000 generations and the parameters were sampled every 10,000 generations. The effective sample size (>200) was determined using Tracer v1.6 and the first 10% of the samples were discarded as burn-in. Tree Annotator v1.8 was used to summarize the set of post burn-in trees and their parameters to produce a maximum clade credibility chronogram showing the mean divergence time estimates with 95% highest posterior density (HPD) intervals. The methodology was adapted from Shahzad et al. (2020). Figtree V.1.4.4 was used to visualize the resulting divergence times.

## 3 Results

### 3.1 Chloroplast genome characteristics

**Figure 1:**
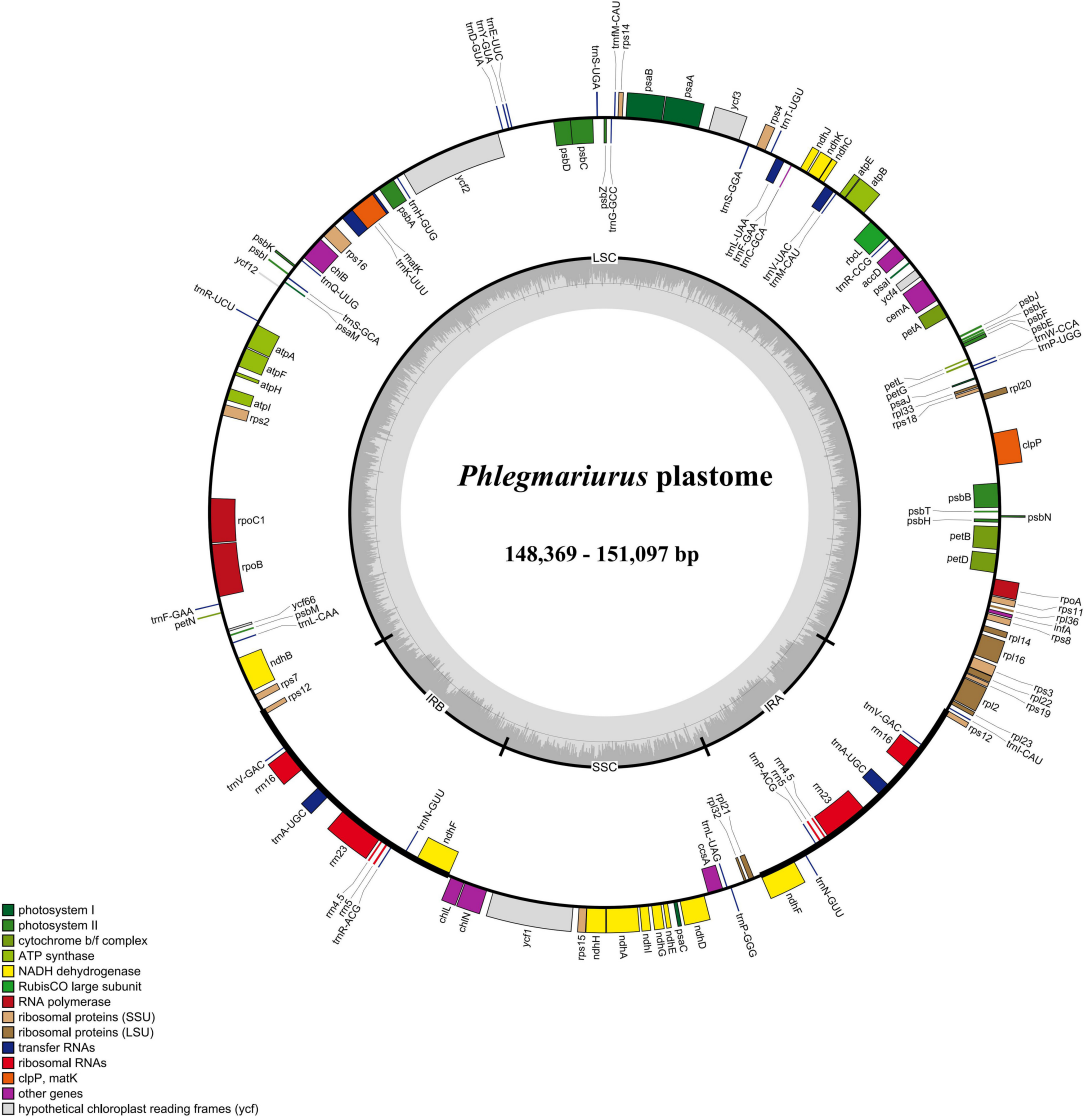
Chloroplast genome map of *Phlegmariurus* species generated in this study. (*Phlegmariurus carinatus* set as an example). The genes inside the outer circle are transcribed clockwise while the outside genes are transcribed anti-clockwise. Genes are color-coded according to their functional groups. The darker gray columns in the inner circle denote the GC content across the genome and the lighter gray columns accordingly correspond to the AT content. IR, inverted repeat; SSC, small single copy; LSC, large single copy. 22 species generated in this study.

The assembled complete chloroplast genomes of *Phlegmariurus* are the typical quadripartite structure (**Figure 1**) composed of one LSC, one SSC, and two IR regions. The total length ranges from 148,369 bp to 151,097 bp including the LSC regions (99,556–100,605 bp), the SSC regions (19,384–19,582 bp), and the IRs (14,702–15,719 bp). The overall GC content is relatively stable (33.8–34.3%) (**Table 2**). We annotated 128 genes in each chloroplast genome with manual checking, including 87 protein-coding genes, 33 transfer RNA (tRNA), and eight ribosomal RNA (rRNA) genes (**Figure 1**; **Tables 1**, **2**). There are two genes including two introns (*clpP* and *ycf3*), nine genes with one intron (*rpl2*, *rpl16*, *petD*, *petB*, *atpF*, *rpoC1*, *ndhB*, *ndhA*, and *rps12*), and there is no intron in the others. The chloroplast genome characteristics of *Phlegmariurus*, such as genome size, gene content, GC content, are summarized in **Table 2**.

**Table 2:** The basic characteristic of the *Phlegmariurus* chloroplast genomes generated in this study.

Species	Size (bp)	LSC (bp)	Ir (bp)	SSC (bp)	GC% content					PCG	tRNA genes	rRNA genes
					Total	Coding	LSC	SSC	IR			
*P. cancellatus*	150823	100186	15576	19485	34.2	35.1	31.9	30.6	43.9	87	33	8
*P. cancellatus*	150719	99998	15619	19483	34.3	35.1	31.9	30.6	43.9	87	33	8
*P. cancellatus*	150062	99573	15452	19582	34.3	35.1	32	30.6	44	87	33	8
*P. carinatus*	149930	100380	15054	19442	33.9	34.8	31.5	30.2	44.3	87	33	8
*P. cryptomerinus*	148629	99605	14806	19412	34	35	31.7	30.4	44.3	87	33	8
*P. cunninghamioides*	150970	100547	15479	19465	33.9	34.8	31.5	30.2	43.8	87	33	8
*P. cunninghamioides*	150865	100380	15054	19442	33.9	34.8	31.5	30.2	44.3	87	33	8
*P. fargesii*	150482	99969	15511	19491	34.3	35.1	31.9	30.6	44	87	33	8
*P. fordii*	150647	100496	15337	19477	33.9	34.8	31.5	30.2	43.9	87	33	8
*P. fordii*	150781	100438	15434	19475	33.9	34.8	31.5	30.2	43.7	87	33	8
*P. fordii*	150464	100401	15300	19463	33.9	34.8	31.5	30.2	43.9	87	33	8
*P. guangdongensis*	150028	100442	14805	19416	33.9	34.9	31.6	30.3	44.2	87	33	8
*P. guangdongensis*	150111	100545	15062	19445	33.9	34.9	31.5	30.3	44.3	87	33	8
*P. guangdongensis*	150643	100526	15338	19441	33.9	34.9	31.5	30.3	43.9	87	33	8
*P. hamiltonii*	148620	99573	14823	19401	34	35	31.7	30.5	44.3	87	33	8
*P. hamiltonii*	148895	99570	14960	19405	34	35	31.7	30.5	44.1	87	33	8
*P. henryi*	150602	100427	15356	19463	33.9	34.8	31.5	30.2	43.9	87	33	8
*P. henryi*	150698	100397	15144	19463	33.9	34.8	31.5	30.3	44.1	87	33	8
*P. henryi*	150148	100509	15431	19461	33.9	34.9	31.5	30.3	43.9	87	33	8
*P. mingcheensis*	148369	99739	14609	19412	34.1	35	31.7	30.1	44.6	87	33	8
*P. obovalifolius*	150466	100265	15366	19469	33.9	34.9	31.6	30.3	43.9	87	33	8
*P. ovatifolius*	148961	99995	14771	19424	34.2	35.1	31.9	30.5	44.5	87	33	8
*P. petiolatus*	148988	100265	15366	19469	34	35	31.6	30.3	43.9	87	33	8
*P. petiolatus*	149047	99708	14965	19409	34	35	31.7	30.4	44.1	87	33	8
*P. petiolatus*	149047	99556	14950	19409	34	35	31.7	30.4	44.1	87	33	8
*P. petiolatus*	148914	99573	14965	19411	34	35	31.7	30.4	44.1	87	33	8
*P. petiolatus*	148758	99557	14899	19403	34	35	31.7	30.4	44.2	87	33	8
*P. petiolatus*	148812	99598	14093	19408	34	35	31.7	30.4	44.2	87	33	8
*P. phlegmaria*	149713	99863	15252	19346	33.8	34.7	31.4	30.2	44	87	33	8
*P. phlegmaria*	149711	99862	15192	19465	33.8	35	31.4	30.1	44	87	33	8
*P. pulcherimus*	148548	99600	14702	19454	34.2	35	31.8	30.5	44.5	87	33	8
*P. pulcherrimus*	148467	99609	14702	19454	34.2	35	31.8	30.5	44.5	87	33	8
*P. salvinioides*	149707	99827	15229	19422	33.8	34.7	31.4	30.1	44	87	33	8
*P. shingianus*	151097	100444	15543	19567	33.9	34.8	31.5	30.3	43.7	87	33	8
*P. sieboldii*	150437	99995	15575	19291	34.2	35.1	31.9	30.6	43.8	87	33	8
*P. sieboldii*	150029	99834	15429	15529	34.2	35	31.8	30.5	44	87	33	8
*P. squarrosus*	150398	100603	15196	19459	34	34.9	31.6	30.3	44.2	87	33	8
*P. squarrosus*	150398	100605	15169	19455	34	34.9	31.6	30.3	44.2	87	33	8
*P. subulifolius*	150612	100460	15295	19562	33.9	34.9	31.5	30.3	44	87	33	8
*P. subulifolius*	150404	100483	15231	19459	33.9	34.9	31.5	30.3	44.1	87	33	8
*P. yunnanensis*	150394	99950	15507	19430	34.3	35.1	32	30.6	44	87	33	8
*P. yunfengii*	150431	100031	15508	19384	34.3	35.1	32	30.3	44	87	33	8

### 3.2 Highly variable regions and repeat sequences

**Figure 2:**
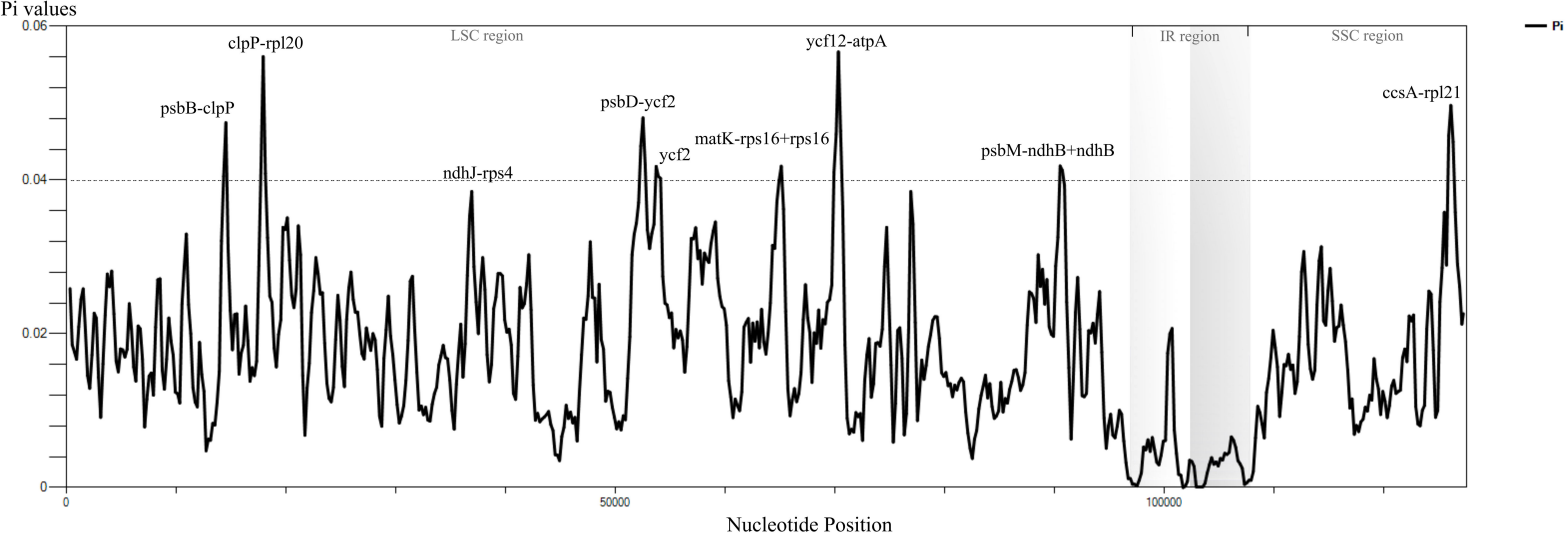
Nucleotide diversity of the entire chloroplast genomes of *Phlegmariurus*. The nucleotide diversity (Pi) by sliding window analysis of *Phlegmariurus* species (window length: 600 bp, step size: 200 bp), which shows Pi values (nucleotide diversity, π) among the complete chloroplast genome sequences. X-axis, the position of the midpoint of each window; Y-axis, nucleotide diversity (π) of each window.

The result of sliding window analysis showed that the sequences of the single copy regions were more variable than those of IR regions (**Figure 2**). Nucleotide diversity (Pi) of the whole chloroplast genome ranged from 0.00008 to 0.00567, with an average of 0.01769 (**Figure 2**; **Supplementary Table S2**). In our results, LSC exhibited the highest (average 0.02090) Pi values, and IR regions exhibited the lowest one (average 0.00440). The regions with Pi values ≥ 0.04 and the aligned length exceeding 600 bp were identified as the divergent hotspots (**Figure 2**; **Supplementary Table S2**). The highest Pi value (0.05668) was found in the *ycf12*–*atpA* region, followed by *clpP*–*rpl20*+*rpl20* (0. 05607), *ccsA*–*rpl21* (0.04972), *psbD*–*ycf*2+*ycf2* (0.04810), *psbB*–*clpP* (0.04747), *psbM*–*ndhB*+*ndhB* (0.04183), and *matK*–*rps16*+*rps16* (0.04181). With the exception of *ccsA–rpl21*, which is located in the SSC region, all other identified regions with high variability are situated in the LSC region (**Figure 2**).

**Figure 3:**
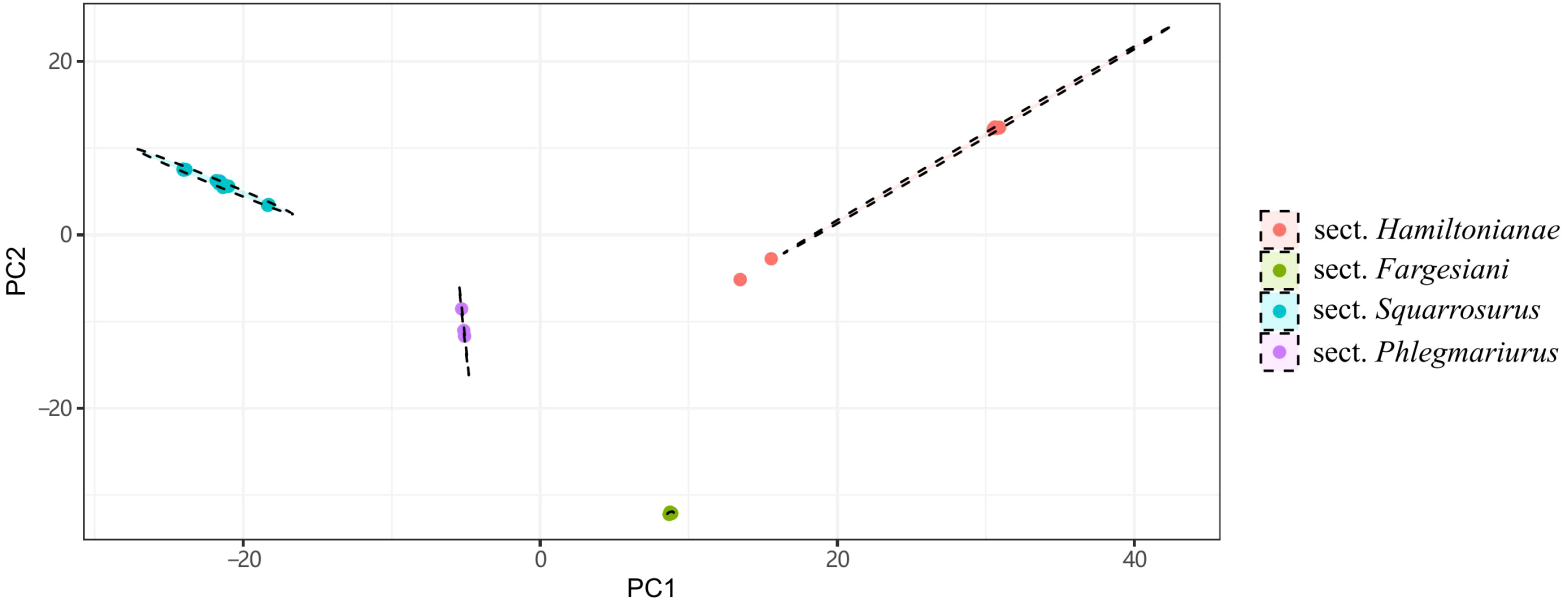
The distribution of four groups on the first 2 discriminant axes of DAPC. Results are generated based on SNPs extracted from the complete chloroplast genomes. Points depicted in different colors represent species belonging to different sections. These cluster colors correspond to the section colors as follows: Sect. *Phlegmariurus* in purple, Sect. *Squarrosurus* in blue, Sect. *Fargesiani* in green, and Sect. *Hamiltonianae* in red.

Ten PCGs with the highest percentage of variable sites are *rps16* (14.39%), *ycf2* (8.67%), *rpl22* (8.33%), *rpl20* (7.54%), *matK* (7.03%), *rps8* (7.02%), *ycf1* (7%), *rpl21* (6.61%), *ycf4* (6.31%), and *cemA* (6.22%) (**Supplementary Figure S1**; **Supplementary Table S3**). The gene *ycf2* (575) had the highest number of parsimony-informative sites, followed by *ycf1* (356), *rpoB* (133), *matK* (119), *cemA* (98), *chlN* (74), *psaA* (68), *rpoC1* (67), *chlB* (62), and *ndhB* (61) (**Supplementary Figure S2**; **Supplementary Table S3**). A total of 10,688 SNPs were identified across the 40 complete chloroplast genomes of *Phlegmariurus*. These SNPs were grouped into four clusters by DAPC, with several outliers observed beyond the 95% confidence ellipse in sect. *Hamiltoniani* (**Figure 3**). The numbers of SSRs ranged from 84 to 125 among *Phlegmariurus* samples. The most common SSR was mono-nucleotide repeats, accounting for about 64.06%, followed by di-nucleotide repeats (ca. 17.53%) (**Supplementary Figure S3**; **Supplementary Table S4**). The four types of SDR and their proportion were forward repeats (F, ca. 44.44%), palindromic repeats (P, ca. 39.81%), reverse repeats (R, ca. 10.1%) and complement repeats (C, ca. 5.65%) (**Supplementary Figure S4**; **Supplementary Table S5**). The number of tandem repeats (TRs) varied from 31 to 56 (**Supplementary Table S6**; **Supplementary Figure S5**).

### 3.3 Positive selection and codon usage analysis

**Figure 4:**
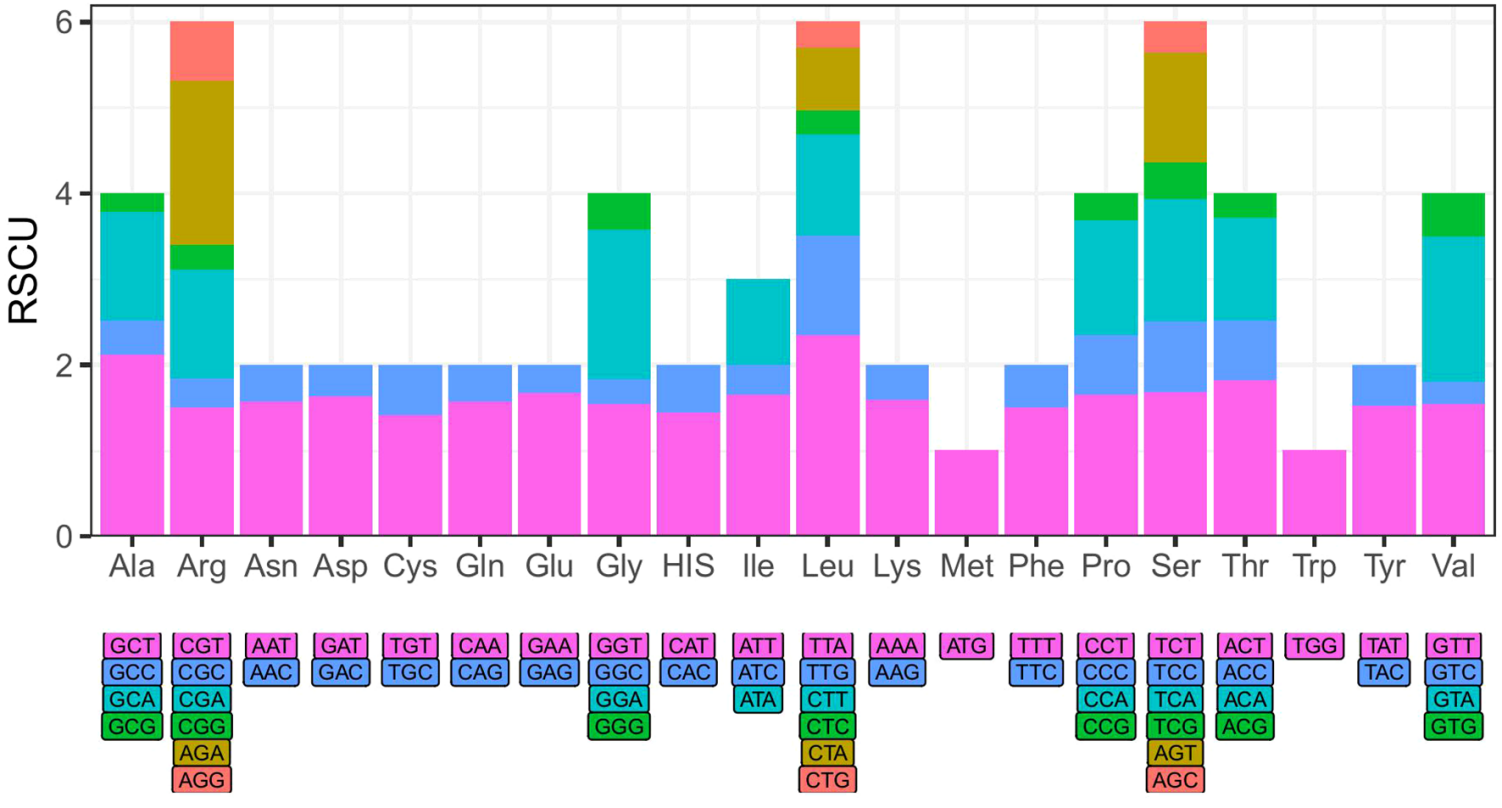
The relative synonymous codon usage (RSCU) of *Phlegmariurus* species calculated based on protein-coding genes from chloroplast genomes. X-axis: Amino acid encoded by different codons; Y-axis: The relative synonymous codon usage (RSCU) values. The color of the histogram is corresponding to the color of codons.

Based on the positive selection analysis of *Phlegmariurus* chloroplast genome protein-coding genes, we identified a total of 12 genes exhibiting signs of positive selection with a significance level of *P* > 0.05 (*atpB*, *cemA*, *chlB*, *chlL*, *chlN*, *ndhB*, *petL*, *psbC*, *psbM*, *rbcL*, *rpoB*, *ycf1*), among which six genes had *P* > 0.01 (*cemA*, *chlB*, *chlN*, *ndhB*, *petL*, *ycf1*) (**Supplementary Tables S7**, **S8**). Notably, the *ycf1* gene exhibited the highest number of positively selected sites, with ten sites detected under the M2 model and 11 sites under the M8 model. The positively selected sites for each gene in detail are listed in **Supplementary Tables S7**, **S8**. These genes encoded three enzyme subunits involved in chlorophyll biosynthesis (*chlB*, *chlL*, chl*N*), two proteins related to photosystem II (*psbC*, *psbM*), as well as proteins associated with ATP subunits, energy production and metabolism, and gene expression and regulation (*atpB*, *cemA*, *ndhB*, *petL*, *rbcL*, *rpoB*, *ycf1*). The codon usage analysis results showed that most amino acids were coded by multiple codons, but arginine (ATG) and tryptophan (TGG) were coded by solitary codon of their own (**Figure 4**).

### 3.4 Molecular phylogenies and divergence time estimation

**Figure 5:**
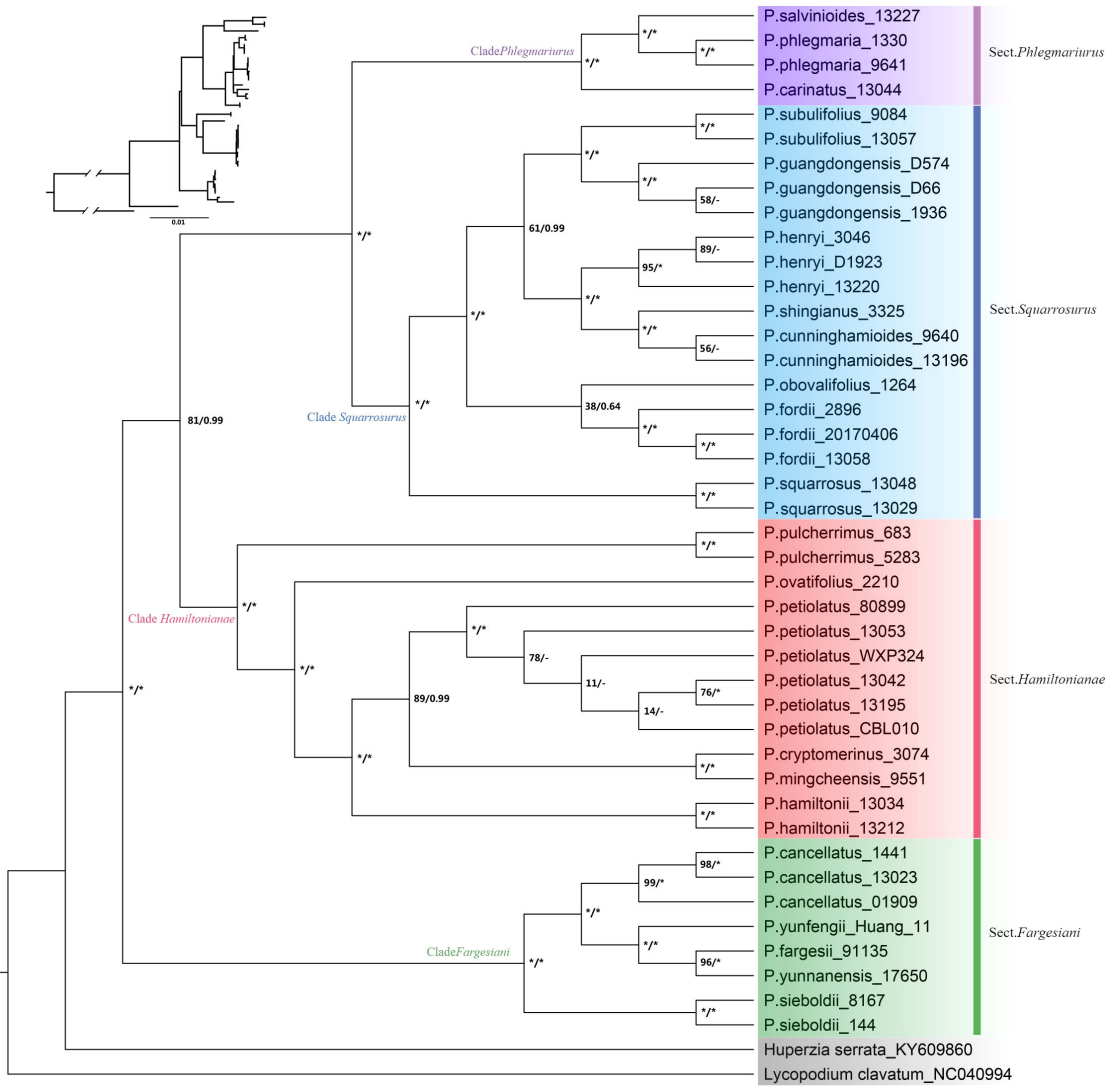
Maximum likelihood (ML) cladogram and phylogram of 42 *Phlegmariurus* samples inferred from the complete chloroplast genomes. ML bootstrap (BS) values and the posterior probabilities (PP) are shown at each node, the asterisk (*) indicates nodes with 100% BS and 1.0 PP. Outgroups are highlighted with gray background. Clade names are represented by different colors background, corresponding to the following sections: Sect. *Phlegmariurus* in purple, Sect. *Squarrosurus* in blue, Sect. *Fargesiani* in green, and Sect. *Hamiltonianae* in red.

**Figure 6:**
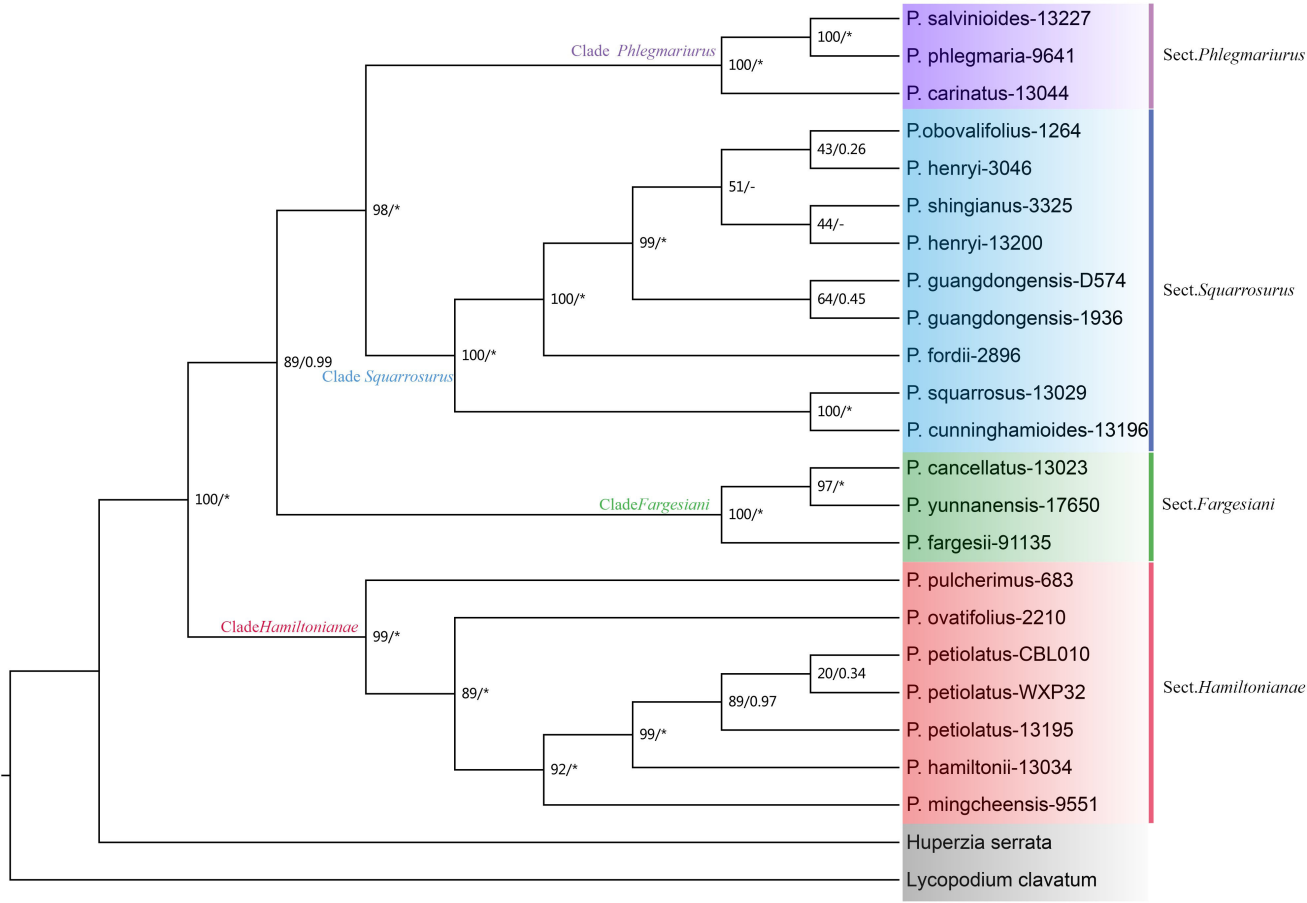
Maximum likelihood (ML) cladogram and phylogram of *Phlegmariurus* inferred from 22 *Phlegmariurus* nuclear ribosomal DNA (nrDNA). ML bootstrap (BS) values and the posterior probabilities (PP) are shown at each node, the asterisk (*) indicates nodes with 100% BS and 1.0 PP. Outgroups are highlighted with gray background. Clade names are represented by different colors background, corresponding to the following sections: Sect. *Phlegmariurus* in purple, Sect. *Squarrosurus* in blue, Sect. *Fargesiani* in green, and Sect. *Hamiltonianae* in red.

The phylogenetic topologies based on both nrDNA and chloroplast genome datasets showed that all the *Phlegmariurus* samples were resolved into four well-supported clades (clade *Fargesiani*, clade *Hamiltoniani*, clade *Phlegmariurus*, and clade *Squarrosurus*), each with strong support (BS ≥ 80%; PP ≥ 0.99) (**Figures 5**, **6**; **Supplementary Figure S6**). The phylogeny based on chloroplast genome showed that clade *Squarrosurus* and clade *Phlegmariurus* were clustered together with strong support (**Figure 5**: BS=100%, PP=1); additionally, clade *Hamiltoniani* was sister to these two aforementioned clades (**Figure 5**: BS=81%, PP=0.99). The clade *Fargesiani* was found to be basal lineage in chloroplast genome phylogeny (**Figure 5**: BS=100%, PP=1). The nrDNA-based phylogenetic result indicated that clade *Squarrosurus* was closely related to clade *Phlegmariurus* (**Figure 6**: BS=98, PP=1). These two clades then clustered with clade *Fargesiani* (**Figure 6**: BS=89, PP=0.99). Additionally, clade *Hamiltoniani* was at the basal position of the genus in nrDNA phylogenetic results (**Figure 6**: BS=100, PP=1).

Based on the three calibration points derived from fossil and second calibration, our analysis estimated that Huperzioideae started to diversify at 84.80 Ma (95% HPD: 50.61–125.42) when *Phylloglossum* diverged from *Huperzia* s.l., a lineage including *Huperzia* and *Phlegmariurus* (**Supplementary Figure S11**). *Huperzia* would have diverged from *Phlegmariurus* around 63.85 Ma (95% HPD: 38.96–91.27). The divergence time of the Paleotropical *Phlegmariurus* in China and neighboring regions was around 26.04 Ma (95% HPD: 14.97–40.01) when sect. *Fargesiani* diverged from the other *Phlegmariurus* linages. Sect. *Hamiltoniani* would have diverged from sect. *Phlegmariurus*-sect. *Squarrosurus* around 23.60 Ma (95% HPD: 13.62–36.43), whereas sect. *Phlegmariurus* was estimated to diverge from sect. *Squarrosurus* around 17.09 Ma (95% HPD: 9.47–28.85).

## 4 Discussion

### 4.1 Chloroplast genome characteristics and potential adaptive selection

The *Phlegmariurus* chloroplast genomes are stable in structure, length, gene content and gene order (**Figure 1**, **Table 2**). The full length was approximately 150K bp (ranging from 148,369 bp to 151,097 bp). The highest GC content (43.7–44.6%) was in the IR regions, while the SSC and LSC regions had lower GC content (30.1–32.0%). This was caused by the total eight rRNAs genes positioned in IR regions in consistent with other plant groups (He et al., 2024; Wang et al., 2024; Qian et al., 2013). In comparison with other genera in Huperzioideae, the genome size differences are minimal: the *Phlegmariurus* is slightly smaller than the *Huperzia* (154,176–154,415 bp; NC_033874; NC_064991) (*H. javanica, H. serrata*), and larger than the *Phylloglossum* (*Phylloglossum drummondii*) (144,520 bp; NC_086515) (Guo et al., 2016; Kwok et al., 2024). Put them in the framework of lycophytes, the chloroplast genomes of the homosporous Huperzioideae display a conserved quadripartite structure with minor sequence variations, however those of the heterosporous Selaginellaceae exhibit highly dynamic structure and extraordinary sequence divergence (Zhang et al., 2019; Kang et al., 2020; Xiang et al., 2022). The sharp contrast phenomena warrants further investigation.

Twelve protein-coding genes are detected under positive selection, most of which are photosynthesis-related (*chlB*, *chlL*, *chlN*, *petL*, *rbcL*, *psbC*, and *psbM*) (**Supplementary Table S7**, **S8**). Given that the *Phlegmariurus* plants grow epiphytically in the forest understory suffering light stress (Jiang and Zhang, 2022; Testo et al., 2018a), we speculate that these photosynthesis-related genes may contribute to light harvesting. Although the function of *ycf* genes remains incompletely understood, the evolutionary significance is well documented (Boudreau and Turmel, 1997). Multiple studies report high nucleotide diversity (*π* values) and accelerated synonymous/nonsynonymous substitution rates of *ycf* genes across plant lineages (Moghaddam et al., 2022; Cho et al., 2024). In *Phlegmariurus*, the situation was in line with other plants that the *ycf1* gene showed the most positively selected sites and ranked seventh in variable site percentage among chloroplast genes (**Supplementary Figure S1**; **Supplementary Table S3**). The *rbcL* gene, encoding the RuBisCO large subunit critical for carbon fixation, has been widely reported to undergo positive selection in land plants (Kapralov and Filatov, 2007). The *chlB*, *chlL*, and *chlN* genes are involved in chlorophyll synthesis, and they are characteristic signature genes in non-seed plants but absent in angiosperms (Mohanta et al., 2020). Their positive selection in *Phlegmariurus* may be critical for photosynthesis and environmental adaptation in these non-seed plants.

### 4.2 Potential association between growth form and chloroplast genomic characteristics

A potential association between different plant growth forms and chloroplast genomic characteristics was proposed recently (Dušková et al., 2010; Bungard, 2004). In parasitic plants, chloroplast genomes are known to undergo gene loss, especially photosynthesis and energy producing related genes, and this phenomenon was regarded as a consequence of adapting to the specialized growth habit (Wicke et al., 2016; Cai et al., 2021; Shen et al., 2020; Xu et al., 2021; Liu et al., 2022). In our study, some *Phlegmariurus* species are obligate epiphytic (species in sect. *Fargesiani*), while the others are facultative (epiphytic and terrestrial; species in sect. *Hamiltoniani*, sect. *Phlegmariurus* and sect. *Squarrosurus*) (Jiang et al., 2023a). However, no much difference in genes and structures of chloroplast genomes was detected between the epiphytic-only and facultative samples. The chloroplast genome variation of parasitic plants would be attributed to the nutritional strategies because they are heterotrophy instead of autotrophy. The genes associated with energy synthesis tend to be lost or become pseudogenized. However, both obligate and facultative epiphytic plants are autotrophic, they retain the crucial genes for photosynthesis under strong selection. This inference is confirmed by the significant positive selection sites detected in photosynthesis-related genes (**Supplementary Tables S7**, **S8**; **Figure 1**).

While the stability of the typical quadripartite structure and gene content in *Phlegmariurus*, there are subtle differences in GC content between species with different growth habits. The GC content of the obligate epiphytic species (sect. *Fargesiani*, 34.2% to 34.3%), seems a little bit higher than that of the facultative species (the other sections) (epiphytic and terrestrial, 33.8% to 34.2%) (**Table 2**). Previous research indicated that monocots in arid environments exhibit higher genomic GC content, indicating a possible link between GC content and environmental stress (Šmarda et al., 2014). This suggests that *Phlegmariurus* may also exhibit similar adaptive evolutionary traits, a possibility that warrants further investigation in future studies.

### 4.3 Potential markers for species identification

Some *Phlegmariurus* species are traditional Chinese herbal medicines, holding considerable potential for extracting the HupA compound (Liu et al., 1986; Yang, 1988b; Ma et al., 2005, 2006; Wu et al., 2011). Previous studies indicated that the HupA content varied among different species; for instance, *P. mingcheensis* yielded higher concentrations at 0.0304%, whereas others, like *P. austrosinicus* (*P. petiolatus*), only at 0.0056% (Yang, 1988b). The interspecific variation in the composition and concentration of medicinal components, coupled with the gross morphological similarities among species, presents a significant challenge for species identification (Ma et al., 2006; Yang, 1988b). It results in confusion among various stakeholders, including traders, pharmaceutical researchers, and consumers. In the other hand, some *Phlegmariurus* species are classified as Vulnerable (VU), while others are listed as Critically Endangered (CR) in the China Plant Red Data Book (https://www.iplant.cn/redbook/splist#CR-PE). Identifying and cultivating specific *Phlegmariurus* species for medicinal resource utilization is crucial. The targeted exploration of these species could significantly diminish the reliance on wild harvesting, which would be instrumental in preserving species diversity and averting its decline (Ma and Gang, 2008; Silalahi et al., 2015).Therefore, the species identification of *Phlegmariurus* is crucial for their utilization and conservation.

The molecular markers used previously, such as *petA*_*trnH*, *rbcL*, *rps4*, *trnL*, *trnL*_*trnF*, and *trnP_petG*, are proven insufficient for the precise identification here, e.g. *P. hamiltonii*, *P. petiolatus*, *P. mingcheensis*, and *P. cryptomerinus* could not be distinguished based on these markers (**Supplementary Figure S12**). Here, we identified hypervariable regions based on the chloroplast genome data as potential markers for future species identification (**Figure 3**). Our results also corroborate the utility of the entire chloroplast genome as a super-marker for species identification (Fu et al., 2019; Krawczyk et al., 2018). This approach successfully distinguished all the *Phlegmariurus* species sampled (**Figures 4**, **5**), established a basis for medicinal material identification.

Lycopodium alkaloid content may vary across the *Phlegmariurus* lineages, with lineage-specific markers identified here enabling species categorization. Geographical variations in alkaloid content (e.g., climatic/geological factors) may existed (Ma et al., 2006; Yang, 1988b), but our uneven sampling (1–6 samples/species) limits the representative in this aspect. Therefore, expanded range-wide sampling is critical to assess medicinal compound variability, refine intraspecies identification, and guide conservation/pharmacological applications.

### 4.4 The phylogenies based on the chloroplast genome and nrDNA and its significance on the infrageneric classification

The phylogenetic results based on the chloroplast genomes and nrDNA data robustly resolved four monophyletic clades within the Chinese *Phlegmariurus*, with each clade containing the same taxa without inter-clade taxonomic inconsistencies (**Figures 3**, **5**, **6**, **Supplementary Figure S6**). As shown in **Figure 5**, the first clade composed of *P. cancellatus*, *P. yunfengii*, *P. fargesii*, *P. yunnanensis* and *P. seiboldii*; the second clade contained *P. hamiltonii*, *P. mingcheensis*, *P. cryptomerinus*, *P. petiolatus*, *P. ovatifolius*, *P. pulcherrimus*; the third clade included *P. squarrosus, P. fordii, P. obovalifolius, P. cunninghamioides, P. shingianus, P. henryi, P. guangdongensis, P. subulifolius* and the fourth clade held *P. carinatus, P. salvinioides*, and *P. phlegmaria* (the type species of *Phlegmariurus*). The SNP-based DAPC results and the number of cpSSRs further corroborated these taxa grouping into the four clusters (**Figures 4**–**6**, **Supplementary Figure S3**).

The relationships among the four clades resolved by nrDNA and chloroplast genome are discordant (**Figures 5**, **6**), except for the sister relationship of clade *Squarrosurus* and clade *Phlegmariurus* which was strongly supported by both datasets (**Figure 4**: BS=100, PP=1; **Figure 5**: BS=98, PP=1). The chloroplast genome resolved clade *Fargesiani* as basal lineage, while the nrDNA recovered clade *Hamiltoniani* as the basal one (**Figures 5**, **6**). The inconsistencies between the nuclear and chloroplast phylogenies could be explained by incomplete lineage sorting, chloroplast capture, hybridization, and introgression events as discussed in numerous angiosperm taxa (Stull et al., 2020; Yang et al., 2021; Acosta and Premoli, 2010; Fehrer et al., 2007). In *Lycopodium sensu lato*, interspecies hybridization turned out to be common especially in species with overlapping geographic range (Xu et al., 2023; Acosta and Premoli, 2010; Wagner and Beitel, 1985; Wagner, 1992). According to the specimen records in PE (Herbarium, Institute of Botany, Chinese Academy of Sciences) and NPSRC (National Plant Specimen Resource Center), most species sampled in this study display overlapping distributions. The discordance between cytoplasmic and nuclear data was often regarded as a result of recurrent hybridization (Petit and Excoffier, 2009; Bouillé et al., 2011; Liu et al., 2022). However, other processes, including incomplete lineage sorting, chloroplast capture, and introgression, may also contribute to these inconsistencies. To elucidate these processes is impossible based on the data here. Further investigation with population sampling and nuclear genome data is necessary to detect the gene flow and clarify the intricate mechanisms. We estimated the divergence times of the *Phlegmariurus* species from China and neighboring regions using protein-coding sequences in complete chloroplast genomes. The diversification between *Huperzia* and *Phlegmariurus* occurred around 63.85 Ma (95% HPD: 38.96–91.27) during the Paleocene. The *Phlegmariurus* in China and neighboring regions diverged around 26.04 Ma (95% HPD: 14.97–40.01) during the Oligocene. This was largely consistent with the previous molecular divergence dating, e.g. the eastern Paleotropical *Phlegmariurus* (including China and neighboring regions) divergence time was around 30 Ma during the Oligocene (Bauret et al., 2018). This study estimated the divergence time of *Phlegmariurus* using all chloroplast protein-coding sequences for the first time.

The resolved four clades well correspond with the four sections recently proposed based on morphological characteristics (Jiang et al., 2023a). Clade *Fargesiani* is consistent with sect. *Fargesiani*, while the remains align with sect. *Hamiltoniani*, sect. *Phlegmariurus*, and sect. *Squarrosurus*, respectively (**Figures 5**, **6**). Historically, the Chinese *Phlegmariurus* classification was subject to several revisions. For instance, the previous sect. *Huperzioides* was subdivided into *s*ect. *Hamiltoniani* and sect. *Squarrosurus* (Zhang and Kung, 1999; 2000; Jiang et al., 2023a). Given the common morphological homoplasy in *Phlegmariurus* (Testo et al., 2018a; Wikström and Kenrick, 2000; Zhang and Kung, 1999; 2000);, the proposed classification systems left uncertainties about the evolutionary coherence of the newly defined sections pending for explicit molecular validation. Here, we provided a comprehensive moleculary phylogenetic examination on the *Phlegmariurus* species in China and neighboring regions. Phylogenetic results of chloroplast and nuclear datasets robustly resolved sect. *Hamiltoniani* and sect. *Squarrosurus* as distinct monophyletic lineages and strongly supported the four sections’ classification (Jiang et al., 2023a). Minor inconsistencies persist between molecular and morphological results. For instance, *P. guangdongensis*, exhibiting significant leaf dimorphism—a trait of sect*. Phlegmariaurus*, was nested within sect. *Squarrosurus* by both chloroplast and nuclear data (**Figures 5**, **6**). Such discrepancies may arise from convergent leaf morphologies driven by similar ecological stress or incomplete lineage sorting. Population-level sampling and nuclear data could construct a more comprehensive evolutionary history of *Phlegmariuru* in future studies.

## 5 Conclusion

The genomic dataset obtained in this study laid a foundation for advancing speciation studies, population genetics, and conservation strategies in *Phlegmariurus*. The 40 *Phlegmariurus* chloroplast genomes we presented and provide critical genetic resources for selecting medicinal species and ornamental variants, offering concrete suggestions for practical applications.

We presented 40 *Phlegmariurus* chloroplast genomes, which serve as a super-marker for species identification and could distinguish all species. These dataset provided critical genetic resources for species identification and breeding of medicinal/ornamental variants. Phylogenetic results robustly validate the four sections’ classification of the Chinese *Phlegmariurus*. Discordance between chloroplast and nrDNA phylogenies suggested complex evolutionary histories in this genus, calling for a further comprehensive integration of single-copy nuclear genes and population sampling to disentangle the underlying mechanisms. The genomic dataset obtained in this study laid a foundation for advancing speciation studies, population genetics, and conservation strategies in *Phlegmariurus*.

## Data Availability

The datasets presented in this study can be found in online repositories. The names of the repository/repositories and accession number(s) can be found below: https://www.ncbi.nlm.nih.gov/genbank/, PP944823–PP944846; https://www.ncbi.nlm.nih.gov/genbank/, PP419991–PP420030.

## Supplementary material

Supplementary Figure 1 Ten most variable sites percentage of protein-coding genes within the assembled *Phlegmariurus* chloroplast genomes.

Supplementary Figure 2 Ten most variable sited number of protein-coding genes within the assembled *Phlegmariurus* chloroplast genomes.

Supplementary Figure 3 Frequency and average proportion of six simple sequence repeats (SSRs) types.

Supplementary Figure 4 Frequency and average proportion of four types of short dispersed repeats (SDRs). Pie chart showing the average proportion of four SDRs types.

Supplementary Figure 5 Analysis of tandem repeats (TRs) in *Phlegmariurus* chloroplast genomes.

Supplementary Figure 6 Maximum likelihood (ML) cladogram of 42 *Phlegmariurus* samples inferred from 87 protein-coding genes in chloroplast genome. ML bootstrap (BS) values are shown at each node.

Supplementary Figure 7 The relative synonymous codon usage (RSCU) of *P. fargesii* calculated based on protein-coding genes.

Supplementary Figure 8 The relative synonymous codon usage (RSCU) of *P. henryi* calculated based on protein-coding genes.

Supplementary Figure 9 The relative synonymous codon usage (RSCU) of *P. hamiltonii* calculated based on protein-coding genes.

Supplementary Figure 10 The relative synonymous codon usage (RSCU) of *P. phlegmaria* calculated based on protein-coding genes.

Supplementary Figure 11 Molecular dating of 21 *Phlegmariurus* species based on the protein-coding sequences in chloroplast genomes.

Supplementary Figure 12 Phylogram based on the plastid sequences published in previous studies by Maximum likelihood (ML). Numbers in each nodes represent ML bootstrap values (BS).

## References

[B1] Acosta M. C. Premoli A. C. (2010). Evidence of chloroplast capture in South American Nothofagus (subgenus Nothofagus, Nothofagaceae). Mol. Phylogenet. Evol. 54, 235–242. doi: 10.1016/j.ympev.2009.08.008, PMID: 19683588

[B2] Amiryousefi A. Jaakko H. Peter P. (2018). IRscope: An online program to visualize the junction sites of chloroplast genomes. Bioinformatics. 17, 17. doi: 10.1093/bioinformatics/bty220, PMID: 29659705

[B3] Bai D. (2007). Development of huperzine A and B for treatment of Alzheimer’s disease. Pure. Appl. Chem. 79, 469–479. doi: 10.1351/pac200779040469

[B4] Bauret L. Field A. R. Gaudeul M. Selosse M. Rouhan G. (2018). First insights on the biogeographical history of *Phlegmariurus* (Lycopodiaceae), with a focus on Madagascar. Mol. Phylogenet. Evol. 127, 488–501. doi: 10.1016/j.ympev.2018.05.004, PMID: 29733977

[B5] Behura S. K. Severson D. W. (2012). Comparative analysis of codon usage bias and codon context patterns between dipteran and hymenopteran sequenced genomes. PloS One 7, e43111. doi: 10.1371/journal.pone.0043111, PMID: 22912801 PMC3422295

[B6] Beier S. Thiel T. Münch T. Scholz U. Mascher M. (2017). MISA–web: a web server for microsatellite prediction. Bioinformatics. 33, 2583–2585. doi: 10.1093/bioinformatics/btx198, PMID: 28398459 PMC5870701

[B7] Benson G. (1999). Tandem repeats finder: a program to analyze DNA sequences. Nucleic Acids Res. 27, 573–580. doi: 10.1093/nar/27.2.573, PMID: 9862982 PMC148217

[B8] Boudreau E. Turmel M. (1997). The *ycf3* and *ycf4* open reading frames are essential for the accumulation of the photosystem I complex in Chlamydomonas reinhardtii. Plant J. 12, 1113–1121. doi: 10.1093/emboj/16.20.6095, PMID: 9321389 PMC1326293

[B9] Bouillé M. Senneville S. Bousquet J. (2011). Discordant mtDNA and cpDNA phylogenies indicate geographic speciation and reticulation as driving factors for the diversification of the genus *Picea* . Tree. Genet. Genomes. 7, 469–484. doi: 10.1007/s11295-010-0349-z

[B10] Bungard R. A. (2004). Photosynthetic evolution in parasitic plants: insight from the chloroplast genome. Bioessays. 26, 235–247. doi: 10.1002/bies.10405, PMID: 14988925

[B11] Cai L. Arnold B. J. Xi Z. Khost D. E. Patel N. Hartmann C. B.. (2021). Deeply altered genome architecture in the endoparasitic flowering plant *Sapria himalayana* Griff. (Rafflesiaceae). Curr. Biol. 31, 1002–1011. doi: 10.1016/j.cub.2020.12.045, PMID: 33485466

[B12] Ching R. C. (1982). The taxonomy of Chinese Lycopodiaceae (sen. lat.) III. Acta Bot. Yunn. 4, 119–128.

[B13] Cho M. S. Yang J. Kim S. H. Crawford D. J. Stuessy T. F. López-Sepúlveda P.. (2024). Plastid phylogenomics of *Robinsonia* (Senecioneae; Asteraceae), endemic to the Juan Fernández Islands: insights into structural organization and molecular evolution. BMC Plant Biol. 24, 1016. doi: 10.1186/s12870-024-05711-3, PMID: 39465373 PMC11514753

[B14] Diego D. Posada D. Kozlov A. M. Stamatakis A. Morel B. Flouri T. (2020). ModelTest-NG: a new and scalable tool for the selection of DNA and protein evolutionary models. Mol. Biol. Evol. 37, 291–294. doi: 10.1093/molbev/msz189, PMID: 31432070 PMC6984357

[B15] Dobrogojski J. Adamiec M. Luciński R. (2020). The chloroplast genome: a review. Acta Physiol. Plant 42, 98. doi: 10.1007/s11738-020-03089-x

[B16] Drummond A. J. Rambaut A. (2007). BEAST: Bayesian evolutionary analysis by sampling trees. BMC Evol. Biol. 7, 214. doi: 10.1186/1471-2148-7-214, PMID: 17996036 PMC2247476

[B17] Dušková E. Kolář F. Sklenář P. Rauchová J. (2010). Genome size correlates with growth form, habitat and phylogeny in the Andean genus *Lasiocephalus* (*Asteraceae*). Preslia. 82, 127–148.

[B18] Fan W. Wu Y. Yang J. Shahzad K. Li Z. (2018). Comparative chloroplast genomics of *Dipsacales* species: Insights into sequence variation, adaptive evolution, and phylogenetic relationships. Front. Plant Sci. 9, 689. doi: 10.3389/fpls.2018.00689, PMID: 29875791 PMC5974163

[B19] Fehrer J. Gemeinholzer B. Chrtek J. Bräutigam S. (2007). Incongruent plastid and nuclear DNA phylogenies reveal ancient intergeneric hybridization in *Pilosella hawkweeds* (Hieracium, Cichorieae, Asteraceae). Mol. Phylogenet. Evol. 42, 347–361. doi: 10.1016/j.ympev.2006.07.004, PMID: 16949310

[B20] Ferreira A. Rodrigues M. Fortuna A. Falcao A. Alves G. (2016). Huperzine A from *Huperzia serrata*:a review of its sources, chemistry, pharmacology and toxicology. Phytochem. Rev. 15, 51–85. doi: 10.1007/s11101-014-9384-y

[B21] Field A. R. Testo W. Bostock P. D. Holtum J. A. M. Waycott M. (2016). Molecular phylogenetics and the morphology of the Lycopodiaceae subfamily Huperzioideae supports three genera: *Huperzia*, *Phlegmariurus* and *Phylloglossum* . Mol. Phylogenet. Evol. 94, 635–657. doi: 10.1016/j.ympev.2015.09.024, PMID: 26493224

[B22] Fu C. N. Wu C. S. Ye L. J. Mo Z. Q. Liu J. Chang Y. W.. (2019). Prevalence of isomeric plastomes and effectiveness of plastome super-barcodes in yews (Taxus) worldwide. Sci. Rep. 9, 2773. doi: 10.1038/s41598-019-39161-x, PMID: 30808961 PMC6391452

[B23] Fu N. Ji M. Rouard M. Wang T. Liu Q. Zhao Z.. (2022). Comparative plastome analysis of Musaceae and new insights into phylogenetic relationships. BMC Genom. 23, 223. doi: 10.1186/s12864-022-08454-3, PMID: 35313810 PMC8939231

[B24] Goldman N. Yang Z. (1994). A codon-based model of nucleotide substitution for protein-coding DNA sequences. Mol. Biol. Evol. 11, 725–736. doi: 10.1093/oxfordjournals.molbev.a040153, PMID: 7968486

[B25] Greiner S. Lehwark P. Bock R. (2019). Organellar Genome DRAW (OGDRAW) version 1.3.1: expanded toolkit for the graphical visualization of organellar genomes. Nucleic. Acids Res. 47, W59–W64. doi: 10.1093/nar/gkz238, PMID: 30949694 PMC6602502

[B26] Grierson J. D. Banks H. P. (1963). Leclercqia complexa gen. et sp. nov., a new lycopod from the late Middle Devonian of New York State. Paleontol. Res. 467, 1–25. doi: 10.1016/0034-6667(72)90005-X

[B27] Guo Z. Y. Zhang H. R. Shrestha N. Zhang X. C. (2016). Complete chloroplast genome of a valuable medicinal plant, *Huperzia serrata* (Lycopodiaceae), and comparison with its congener. Appl. Plant Sci. 4, apps.1600071. doi: 10.3732/apps.1600071, PMID: 27843724 PMC5104525

[B28] He M. Han X. Qin X. Bao J. Li H. Xie Q.. (2024). Comparative chloroplast genome analyses provide new insights into phylogeny of *Taraxacum* and molecular markers for distinguishing rubber producing dandelions from their weedy relatives in China. Ind. Crops. Prod. 207, 117712. doi: 10.1016/j.indcrop.2023.117712

[B29] Jiang R. H. Liang S. Q. Wu F. Tang L. M. Qin B. Chen Y. Y.. (2023b). Phylogenomic analysis, cryptic species discovery, and DNA barcoding of the genus *Cibotium* in China based on plastome data. Front. Plant Sci. 14, 1183653. doi: 10.3389/fpls.2023.1183653, PMID: 37346120 PMC10279961

[B30] Jiang R. H. Xiang R. C. Zhang X. C. (2023a). A taxonomie revision of *Phlegmariurus* Holub (Lyeopodiaceae) from China. Guihaia. 43, 1759–1783. doi: 10.11931/guihaia.gxzw202201049

[B31] Jiang R. H. Zhang X. C. (2022). Two new species of *Phlegmariurus* from China. Turczaninowia. 25, 129–134. doi: 10.14258/turczaninowia.25.1.11

[B32] Jin J. J. Yu W. B. Yang J. B. Song Y. Pamphilis C. W. Yi T. S.. (2020). GetOrganelle: A fast and versatile toolkit for accurate *de novo* assembly of organelle genomes. Genome Biol. 21, 241. doi: 10.1186/s13059-020-02154-5, PMID: 32912315 PMC7488116

[B33] Jombart T. (2008). adegenet:a R package for the multivariate analysis of genetic markers. Bioinformatics. 24, 1403–1405. doi: 10.1093/bioinformatics/btn129, PMID: 18397895

[B34] Jombart T. Ahmed I. (2011). adegenet 1.3-1: new tools for the analysis of genome–wide SNP data. Bioinformatics. 27, 3070–3071. doi: 10.1093/bioinformatics/btr521, PMID: 21926124 PMC3198581

[B35] Jombart T. Devillard S. Balloux F. (2010). Discriminant analysis of principal components:a new method for the analysis of genetically structured populations. BMC Genet. 11, 94. doi: 10.1186/1471-2156-11-94, PMID: 20950446 PMC2973851

[B36] Kalyaanamoorthy S. Minh B. Q. Wong T. K. Haeseler A. Jermiin L. S. (2017). ModelFinder: fast model selection for accurate phylogenetic estimates. Nat. Methods 14, 587–589. doi: 10.1038/nmeth.4285, PMID: 28481363 PMC5453245

[B37] Kang J. S. Zhang H. R. Wang Y. R. Liang S. Q. Mao Z. Y. Zhang X. C.. (2020). Distinctive evolutionary pattern of organelle genomes linked to the nuclear genome in Selaginellaceae. Plant J. 104, 1657–1672. doi: 10.1111/tpj.v104.6, PMID: 33073395

[B38] Kapralov M. V. Filatov D. A. (2007). Widespread positive selection in the photosynthetic Rubisco enzyme. BMC Evol. Biol. 7, 72. doi: 10.1186/1471-2148-7-73, PMID: 17498284 PMC1884142

[B39] Katoh K. Standley D. M. (2013). MAFFT Multiple Sequence Alignment Software Version 7: Improvements in performance and usability. Mol. Biol. Evol. 30, 772–780. doi: 10.1093/molbev/mst010, PMID: 23329690 PMC3603318

[B40] Kearse M. Moir R. Wilson A. Stones-Havas S. Cheung M. Sturrock S.. (2012). Geneious Basic: an integrated and extendable desktop software platform for the organization and analysis of sequence data. Bioinformatics. 28, 1647–1649. doi: 10.1093/bioinformatics/bts199, PMID: 22543367 PMC3371832

[B41] Kozlov A. M. Darriba D. Flouri T. Morel B. Stamatakis A. (2019). RAxML-NG: A fast, scalable, and user–friendly tool for maximum likelihood phylogenetic inference. Bioinformatics. 35, 4453–4455. doi: 10.1093/bioinformatics/btz305, PMID: 31070718 PMC6821337

[B42] Krawczyk K. Nobis M. Myszczynski K. Klichowska E. Sawicki J. (2018). Plastid super-barcodes as a tool for species discrimination in feather grasses (Poaceae: *Stipa*). Sci. Rep. 8, 1924. doi: 10.1038/s41598-018-20399-w, PMID: 29386579 PMC5792575

[B43] Kurtz S. Choudhuri J. V. Ohlebusch E. Schleiermacher C. Stoye J. Giegerich R.. (2001). REPuter: the manifold applications of repeat analysis on a genomic scale. Nucleic. Acids Res. 29, 4633–4642. doi: 10.1093/nar/29.22.4633, PMID: 11713313 PMC92531

[B44] Kwok G. F. M. Viljoen A. Campbell-Clause L. Smith J. Jones A. Brown P.. (2024). Insights into U-to-C RNA editing from the lycophyte. Phylloglossum drummondii. Plant J. 119, 445–459. doi: 10.1111/tpj.16775, PMID: 38652016

[B45] Little J. T. Walsh S. Aisen P. S. (2008). An update on huperzine A as a treatment for Alzheimer’s disease. Expert. Opin. Invest. Drugs 17, 209–215. doi: 10.1517/13543784.17.2.209, PMID: 18230054

[B46] Liu J. S. Yu C. M. Zhou Y. Z.. (1986). Study on the chemistry of huperzine–A and huperzine-B. Acta Chim. Sinica. 44, 1035–1040.

[B47] Luo T. Li Y. Yuan X. Wang Y. (2019). The complete chloroplast genome sequence of *Phlegmariurus carinatus* . Mitochondrial DNA B. 5, 3418–3419. doi: 10.1080/23802359.2019.1688720, PMID: 33366278 PMC7707723

[B48] Ma X. Gang D. R. (2008). *In vitro* production of huperzine A, a promising drug candidate for Alzheimer’s disease. Phytochemistry. 69, 2022–2028. doi: 10.1016/j.phytochem.2008.04.017, PMID: 18538805

[B49] Ma X. G. Ren Y. B. Sun H. (2024). Introgression and incomplete lineage sorting blurred phylogenetic relationships across the genomes of sclerophyllous oaks from southwest China. Cladistics. 40, 357–373 .doi: 10.1111/cla.12570, PMID: 38197450

[B50] Ma X. Tan C. Zhu D. Gang D. R. (2005). Is there a better source of huperzine A than *Huperzia serrata*? Huperzine A content of Huperziaceae species in China. J. Agric. Food. Chem. 53, 1393–1398. doi: 10.1021/jf048193n, PMID: 15740012

[B51] Ma X. Tan C. Zhu D. Gang D. R. (2006). A survey of potential huperzine A natural resources in China: the Huperziaceae. J. Ethnopharmacol. 104, 54–67. doi: 10.1016/j.jep.2005.08.042, PMID: 16203116

[B52] Moghaddam M. Ohta A. Shimizu M. Terauchi R. Kazempour S. (2022). The complete chloroplast genome of *Onobrychis gaubae* (Fabaceae-Papilionoideae): comparative analysis with related IR-lacking clade species. BMC Plant Biol. 22, 75. doi: 10.1186/s12870-022-03465-4, PMID: 35183127 PMC8858513

[B53] Mohanta T. K. Mishra A. K. Khan A. Hashem A. Allah E. F. A. Al-Harrasi A.. (2020). Gene loss and evolution of the plastome. Genes. 11, 1133. doi: 10.3390/genes11101133, PMID: 32992972 PMC7650654

[B54] National Plant Specimen Resource Center National Plant Specimen Resource Center information network. Available online at: https://www.plantplus.cn/cn/news/24. (Accessed September 20, 2024).

[B55] Nett R. S. Dhoc Y. Low Y. Y. Sattely E. S. (2021). A metabolic regulon reveals early and late acting enzymes in neuroactive Lycopodium alkaloid biosynthesis. Proc. Natl. Acad. Sci. U.S.A. 118, e2102949118. doi: 10.1073/pnas.2102949118, PMID: 34112718 PMC8214681

[B56] Petit R. J. Excoffier L. (2009). Gene flow and species delimitation. Trends Ecol. Evol. 24, 386–393. doi: 10.1016/j.tree.2009.02.011, PMID: 19409650

[B57] PPGI (2016). A community–derived classification for extant lycophytes and ferns. J. Syst. Evol. 54, 563–603. doi: 10.1111/jse.12229

[B58] Qian J. Song J. Y. Gao H. H. Zhu Y. J. Xu J. Pang X. H.. (2013). The complete chloroplast genome sequence of the medicinal plant *Salvia miltiorrhiza* . Plos. One 8, e57607. doi: 10.1371/journal.pone.0057607, PMID: 23460883 PMC3584094

[B59] Ronquist F. Huelsenbeck J. P. (2003). MrBayes 3: Bayesian phylogenetic inference under mixed models. Bioinformatics. 19, 1572–1574. doi: 10.1093/bioinformatics/btg180, PMID: 12912839

[B60] Rozas J. Ferrer–Mata A. Sanchez–DelBarrio J. C. Guirao-Rico S. Librado P. Ramos-Onsins S. E.. (2017). DnaSP 6: DNA sequence polymorphism analysis of large data sets. Mol. Biol. Evol. 34, 3299–3302. doi: 10.1093/molbev/msx248, PMID: 29029172

[B61] Shahzad K. Liu M. Zhao Y. Zhang T. Liu J. Li Z.. (2020). Evolutionary history of endangered and relict tree species *Dipteronia sinensis* in response to geological and climatic events in the Qinling Mountains and adjacent areas. Ecol. Evol. 10, 14052–14066. doi: 10.1002/ece3.v10.24, PMID: 33391701 PMC7771168

[B62] Shen G. Liu N. Zhang J. Xu Y. Baldwin L. Wu J. (2020). *Cuscuta australis* (dodder) parasite eavesdrops on the host plants’ FT signals to flower. Proc. Natl. Acad. Sci. 117, 23125–23130. doi: 10.1073/pnas.2009445117, PMID: 32868415 PMC7502711

[B63] Silalahi M. Nisyawati Walujo E. B. Supriatna J. Mangunwardoyo W. (2015). The local knowledge of medicinal plants trader and diversity of medicinal plants in the Kabanjahe traditional market, North Sumatra, Indonesia. J. Ethnopharmacol. 175, 432–443. doi: 10.1016/j.jep.2015.09.009, PMID: 26435224

[B64] Šmarda P. Bureš P. Horová L. Leitch I. J. Mucina L. (2014). Ecological and evolutionary significance of genomic GC content diversity in monocots. Proc. Natl. Acad. Sci. U. S. A. 111, E4096–E4102. doi: 10.1073/pnas.1321152111, PMID: 25225383 PMC4191780

[B65] State Forestry and Grassland Administration and the Ministry of Agriculture and Rural Affairs, P. R. China (2021). List of wild plants under state protection. Available online at: http://www.forestry.gov.cn/main/5461/20210908/162515850572900.html. (Accessed September 20, 2024).

[B66] Stull G. W. Soltis P. S. Soltis D. E. Gitzendanner M. A. Smith S. A. (2020). Nuclear phylogenomic analyses of asterids conflict with plastome trees and support novel relationships among major lineages. Am. J. Bot. 107, 790–805. doi: 10.1002/ajb2.v107.5, PMID: 32406108

[B67] Tamura K. Stecher G. Kumar S. (2021). MEGA11: molecular evolutionary genetics analysis version 11. Mol. Biol. Evol. 38, 3022–3027. doi: 10.1093/molbev/msab120, PMID: 33892491 PMC8233496

[B68] Tang L. M. Jiang R. H. An J. C. (2020). The complete chloroplast genome of *Phlegmariurus phlegmaria*, one representative species of genus *Phlegmariurus* . Mitocondrial. DNA. B. 3, 3148–3419. doi: 10.1080/23802359.2020.1820392, PMID: 33458191 PMC7782255

[B69] Testo W. Field A. Barrett D. S. (2018b). Overcoming among-lineage rate heterogeneity to infer the divergence times and biogeography of the clubmoss family Lycopodiaceae. J. Biogeography. 45, 1929–1941. doi: 10.1111/jbi.2018.45.issue-8

[B70] Testo W. Øllgaard B. Field A. Almeida T. Kessler M. Barrington D. (2018a). Phylogenetic systematics, morphological evolution, and natural groups in neotropical *Phlegmariurus* (Lycopodiaceae). Mol. Phylogenet. Evol. 125, 1–13. doi: 10.1016/j.ympev.2018.03.016, PMID: 29559245

[B71] Wagner F. S. (1992). Cytological problems in *lycopodium* sens. Lat. Ann. Mo. Bot. Gard. 79, 718–729. doi: 10.2307/2399761

[B72] Wagner F. S. Beitel J. M. (1985). “Evidence for interspecific hybridization pteridophytes with subterranean mycoparasitic gametophytes,” in Biology of pteridophytes. Proc. Roy. Soc. Edinburgh, vol. 86B . Eds. Dyer A. F. Page C. N., 273–281. doi: 10.1017/S026972700000823X

[B73] Wang G. Ren Y. Su Y. Zhang H. Li J. Han J.. (2024). Molecular marker development and phylogenetic analysis of *Aconitum* species based on chloroplast genomes. Ind. Crops. Prod. 221, 119386. doi: 10.1016/j.indcrop.2024.119386

[B74] Wei R. Yan Y. H. Harris A. J. Kang J. S. Shen H. Xiang Q. P.. (2017). Plastid phylogenomics resolve deep relationships among eupolypod II ferns with rapid radiation and rate heterogeneity. Genome. Biol. Evol. 9, 1646–1657. doi: 10.1093/gbe/evx107, PMID: 28854625 PMC5534337

[B75] Wei R. Yang J. He L. J. Liu H. M. Hu J. Y. Liang S. Q.. (2021). Plastid phylogenomics provides novel insights into the infrafamilial relationship of Polypodiaceae. Cladistics. 37, 717–727. doi: 10.1111/cla.12461, PMID: 34841589

[B76] Wei R. Zhang X. C. (2020). Phylogeny of *Diplazium* (Athyriaceae) revisited: Resolving the backbone relationships based on plastid genomes and phylogenetic tree space analysis. Mol. Phylogenet. Evol. 143, 106699. doi: 10.1016/j.ympev.2019.106699, PMID: 31809851

[B77] Wei R. Zhang X. C. (2022). A revised subfamilial classification of Polypodiaceae based on plastome, nuclear ribosomal, and morphological evidence. Taxon. 2, 71. doi: 10.1002/tax.12658

[B78] Wicke S. Müller K. F. Pamphilis C. W. Quandt D. Bellot S. Schneeweiss G. M. (2016). Mechanistic model of evolutionary rate variation en route to a nonphotosynthetic lifestyle in plants. Proc. Natl. Acad. Sci. 113, 9045–9050. doi: 10.1073/pnas.1607576113, PMID: 27450087 PMC4987836

[B79] Wikström N. Kenrick P. (1997). Phylogeny of Lycopodiaceae (Lycopsida) and the relationships of *Phylloglossum drummondii* Kunze based on *rbcL* sequences. Int. J. Plant Sci. 158, 862–871. doi: 10.1086/297501

[B80] Wikström N. Kenrick P. (2000). Phylogeny of epiphytic *Huperzia* (Lycopodiaceae): Paleotropical and Neotropical clades corroborated by *rbcL* sequences. Nord. J. Bot. 20, 165–171. doi: 10.1111/j.1756-1051.2000.tb01561.x

[B81] Wikström N. Kenrick P. (2001). Evolution of Lycopodiaceae (Lycopsida): estimating divergence times from *rbcL* gene sequences by use of nonparametric rate smoothing. Mol. Phylogenet. Evol. 19, 177–186. doi: 10.1006/mpev.2001.0936, PMID: 11341801

[B82] Wikström N. Kenrick P. Chase M. (1999). Epiphytism and terrestrialization in tropical *Huperzia* (Lycopodiaceae). Plant Syst. Evol. 218, 221–243. doi: 10.1007/BF01089229

[B83] Wingett S. W. Andrews S. (2018). FastQ screen: a tool for multi-genome mapping and quality control. F1000Research. 7, 1338. Available at: http://www.bioinformatics.babraham.ac.uk/projects/fastqc/., PMID: 30254741 10.12688/f1000research.15931.1 PMC6124377

[B84] Wolf P. G. Karol K. G. Mandoli D. F. Kuehl J. Arumuganathan K. Ellis M. W.. (2005). The first complete chloroplast genome sequence of a lycophyte, *Huperzia lucidula* (Lycopodiaceae). Gene. 350, 117–128. doi: 10.1016/j.gene.2005.01.018, PMID: 15788152

[B85] Wu T. Y. Chen C. P. Chen C. P. Jin T. R. (2011). Traditional Chinese medicines and Alzheimer’s disease. Taiwan. J. Obs. Gynecol. 50, 131–135. doi: 10.1016/j.tjog.2011.04.004, PMID: 21791295

[B86] Xiang Q. P. Tang J. Y. Yu J. G. Smith D. R. Zhu Y. M. Wang Y. R.. (2022). The evolution of extremely diverged plastomes in Selaginellaceae (lycophyte) is driven by repeat patterns and the underlying DNA maintenance machinery. Plant J. 111, 768–784. doi: 10.1111/tpj.v111.3, PMID: 35648423

[B87] Xu Z. L. Chu B. M. Luan X. H. Wu W. T. Cai D. G. (1985). Structural determination of fordine. Mil. Med. Res. 03, 222.

[B88] Xu T. Zhang J. Ma C. Lei Y. Shen G. Jin J.. (2022). Comparative genomics of orobanchaceous species with different parasitic lifestyles reveals the origin and stepwise evolution of plant parasitism. Mol. Plant 15, 1384–1399. doi: 10.1016/j.molp.2022.07.007, PMID: 35854658

[B89] Xu M. Heidmarsson S. Boer H. J. Kool A. Olafsdottir E. S. (2019). Ethnopharmacology of the club moss subfamily Huperzioideae (Lycopodiaceae, Lycopodiophyta): A phylogenetic and chemosystematic perspective. J. Ethnopharmacol. 245, 112130. doi: 10.1016/j.jep.2019.112130, PMID: 31376517

[B90] Xu W. Lu R. Li J. Xia M. Chen G. Li P.. (2023). Comparative plastome analyses and evolutionary relationships of all species and cultivars within the medicinal plant genus *Atractylodes* . Ind. Crops. Prod. 201, 116974. doi: 10.1016/j.indcrop.2023.116974

[B91] Xu Y. Yang Z. (2013). PAMLX: A graphical user interface for PAML. Mol. Biol. Evol. 30, 2723–2724. doi: 10.1093/molbev/mst179, PMID: 24105918

[B92] Xu Y. Zhang J. Ma C. Zhao M. Zhang J. Shen G.. (2021). A chromosome–scale Gastrodia elata genome and large–scale comparative genomic analysis indicate convergent evolution by gene loss in mycoheterotrophic and parasitic plants. Plant J. 108, 1609–1623. doi: 10.1111/tpj.v108.6, PMID: 34647389

[B93] Yang C. Y. (1988a). Medicinal plants of the *phlegmariurus* in China. Chin. Herb. Med. 3, 36–38.

[B94] Yang C. Y. (1988b). An overview of plant resources and pharmacological effects of fordine. J. Chin. Mater. Med. 1, 40–42.

[B95] Yang C. Y. (1990). Revision of lycopodiales from China. Bull. Acad. Milit Med. Sci.1990; 14, 269–275.

[B96] Yang Z. Bielawski J. P. (2000). Statistical methods for detecting molecular adaptation. Trends. Ecol. Evol. 15, 496–503. doi: 10.1016/S0169-5347(00)01994-7, PMID: 11114436 PMC7134603

[B97] Yang Z. Nielsen R. Goldman N. Pedersen A. M. (2000). Codon-substitution models for heterogeneous selection pressure at amino acid sites. Genetics. 155, 431–448. doi: 10.1093/genetics/155.1.431, PMID: 10790415 PMC1461088

[B98] Yang Y. Y. Qu X. J. Zhang R. Stull G. W. Yi T. S. (2021). Plastid phylogenomic analyses of Fagales reveal signatures of conflict and ancient chloroplast capture. Mol. Phylogenet. Evol. 163, 107232. doi: 10.1016/j.ympev.2021.107232, PMID: 34129935

[B99] Yang Z. Nielsen R. Goldman N. Pedersen A. M. (2005). Bayes empirical Bayes inference of amino acid sites under positive selection. Mol. Biol. Evol. 22, 1107–1117. doi: 10.1093/molbev/msi097, PMID: 15689528

[B100] Yang J. Xiang Q. P. Zhang X. C. (2022). Uncovering the hidden diversity of the rosette–forming *Selaginella tamariscina* group based on morphological and molecular data. Taxon. 72, 8–19. doi: 10.1002/tax.12817

[B101] Zhang L. B. Kung H. S. (1999). On the taxonomy of *Phlegmariurus* (Herter) Holub sect. Huperzioides H.S. Kung et L.B. Zhang (sect. nov.) with notes on the infrageneric classification of the genus *Phlegmariurus* in China. J. Syst. Evol. 37, 40–53.

[B102] Zhang L. B. Kung H. S. (2000). Two sections of *phlegmariurus* (Herter) holub (Huperziaceae) from China. J. Syst. Evol. 38, 23–29.

[B103] Zhang H. R. Wei R. Xiang Q. P. Zhang X. C. (2020). Plastome-based phylogenomics resolves the placement of the *sanguinolenta* group in the spikemoss of lycophyte (Selaginellaceae). Mol. Phylogenet. Evol. 147, 106788. doi: 10.1016/j.ympev.2020.106788, PMID: 32173413

[B104] Zhang H. R. Xiang Q. P. Zhang X. C. (2019). The unique evolutionary trajectory and dynamic conformations of DR and IR/DR-coexisting plastomes of the early vascular plant Selaginellaceae (Lycophyte). Genome. Biol. Evol. 11, 1258–1274. doi: 10.1093/gbe/evz073, PMID: 30937434 PMC6486807

[B105] Zhang M. H. Xiang Q. P. Zhang X. C. (2022). Plastid phylogenomic analyses of the *Selaginella sanguinolenta* group (Selaginellaceae) reveal conflict signatures resulting from sequence types, outlier genes, and pervasive RNA editing. Mol. Phylogenet. Evol. 173, 107507. doi: 10.1016/j.ympev.2022.107507, PMID: 35589053

[B106] Zhou J. Zhang H. Y. Tang X. C. (2001). Huperzine A attenuates cognitive deficits and hippocampal neuronal damage after transient global ischemia in gerbils. Neurosci. Lett. 313, 137–140. doi: 10.1016/S0304-3940(01)02265-0, PMID: 11682146

