# Supplementary figures and images for: Comparative chloroplast genomes and phylogenetic analysis of the Phlegmariurus (Lycopodiaceae) from China and neighboring regions
Source: Front Plant Sci. 2025 Jul 8;16:1543431. doi: 10.3389/fpls.2025.1543431 (PMC12279849; doi:10.3389/fpls.2025.1543431)

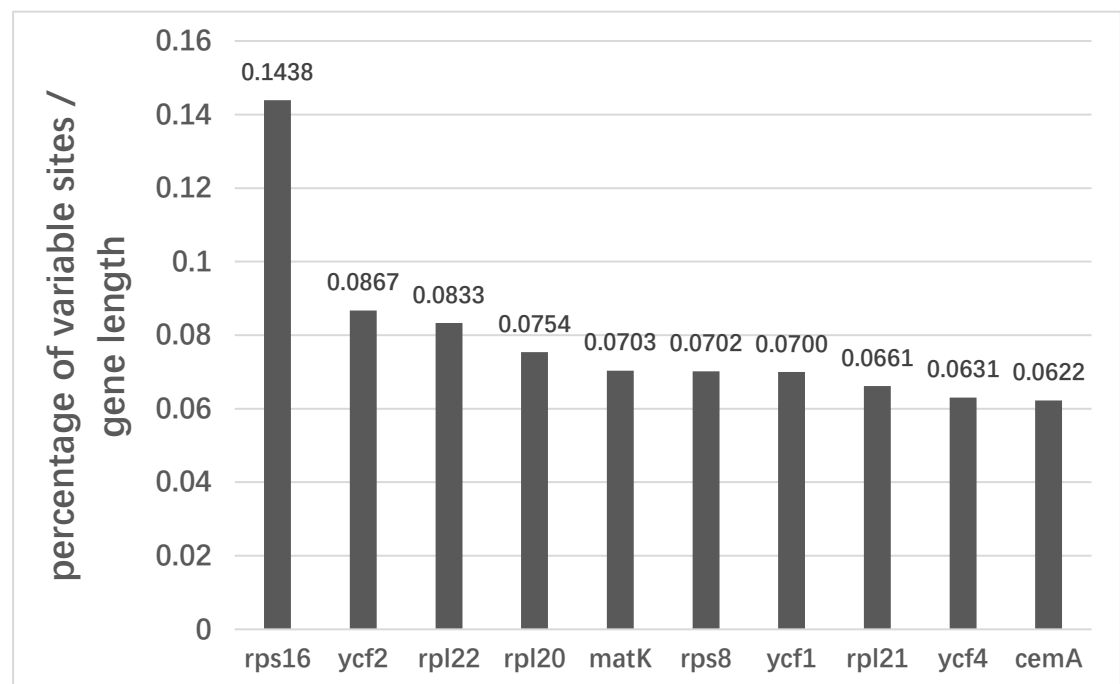

Supplement: Supplementary Figure 1 — Ten most variable sites percentage of protein-coding genes within the assembled Phlegmariurus chloroplast genomes. [file Image1.pdf]

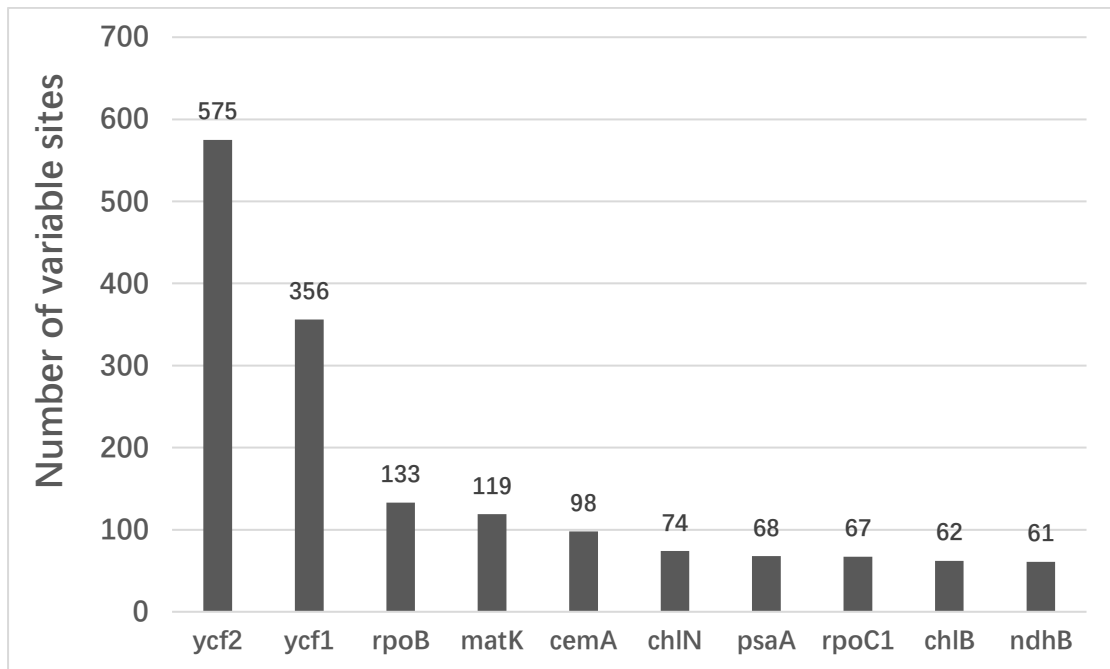

Supplement: Supplementary Figure 2 — Ten most variable sited number of protein-coding genes within the assembled Phlegmariurus chloroplast genomes. [file Image2.pdf]

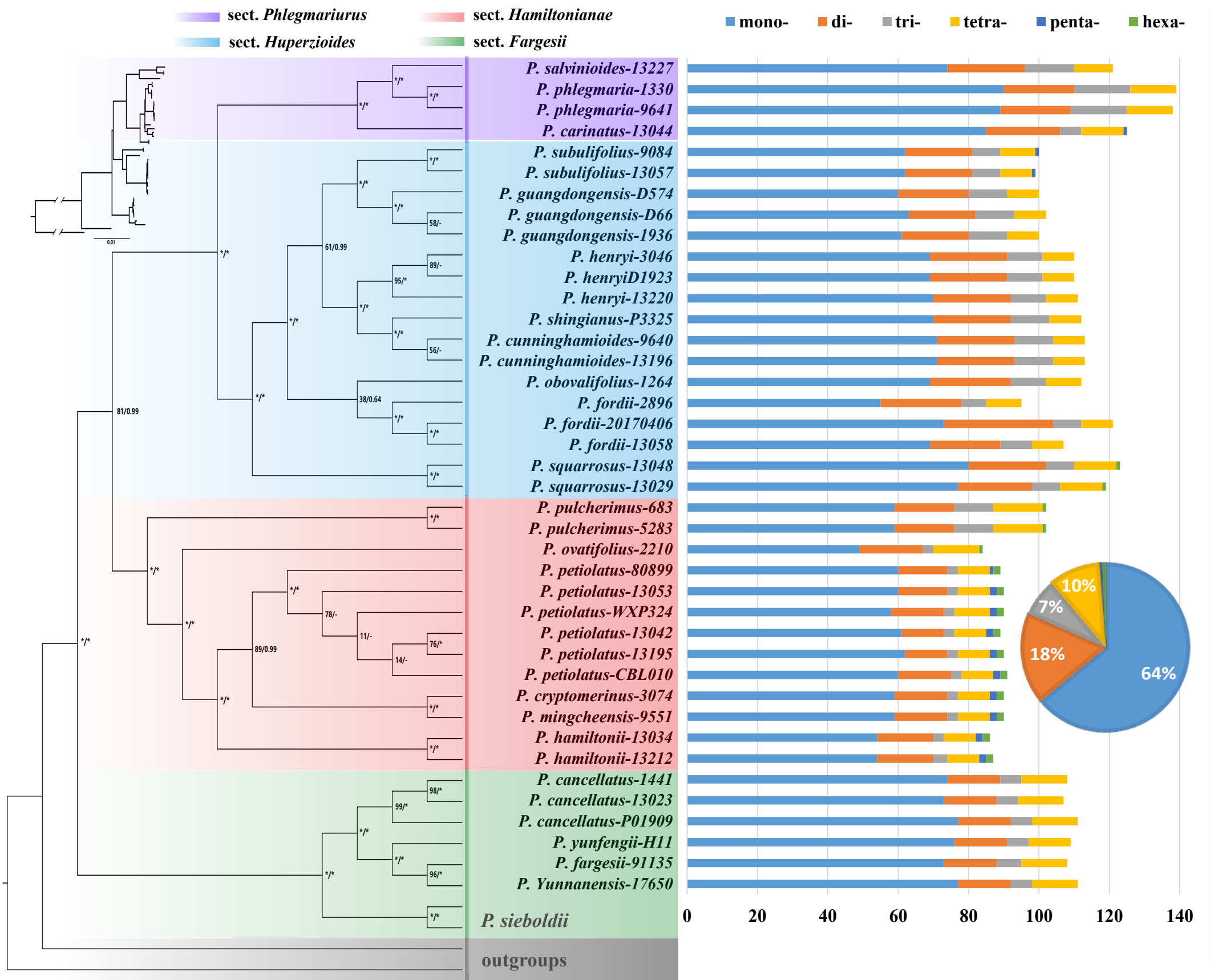

Supplement: Supplementary Figure 3 — Frequency and average proportion of six simple sequence repeats (SSRs) types. [file Image3.pdf]

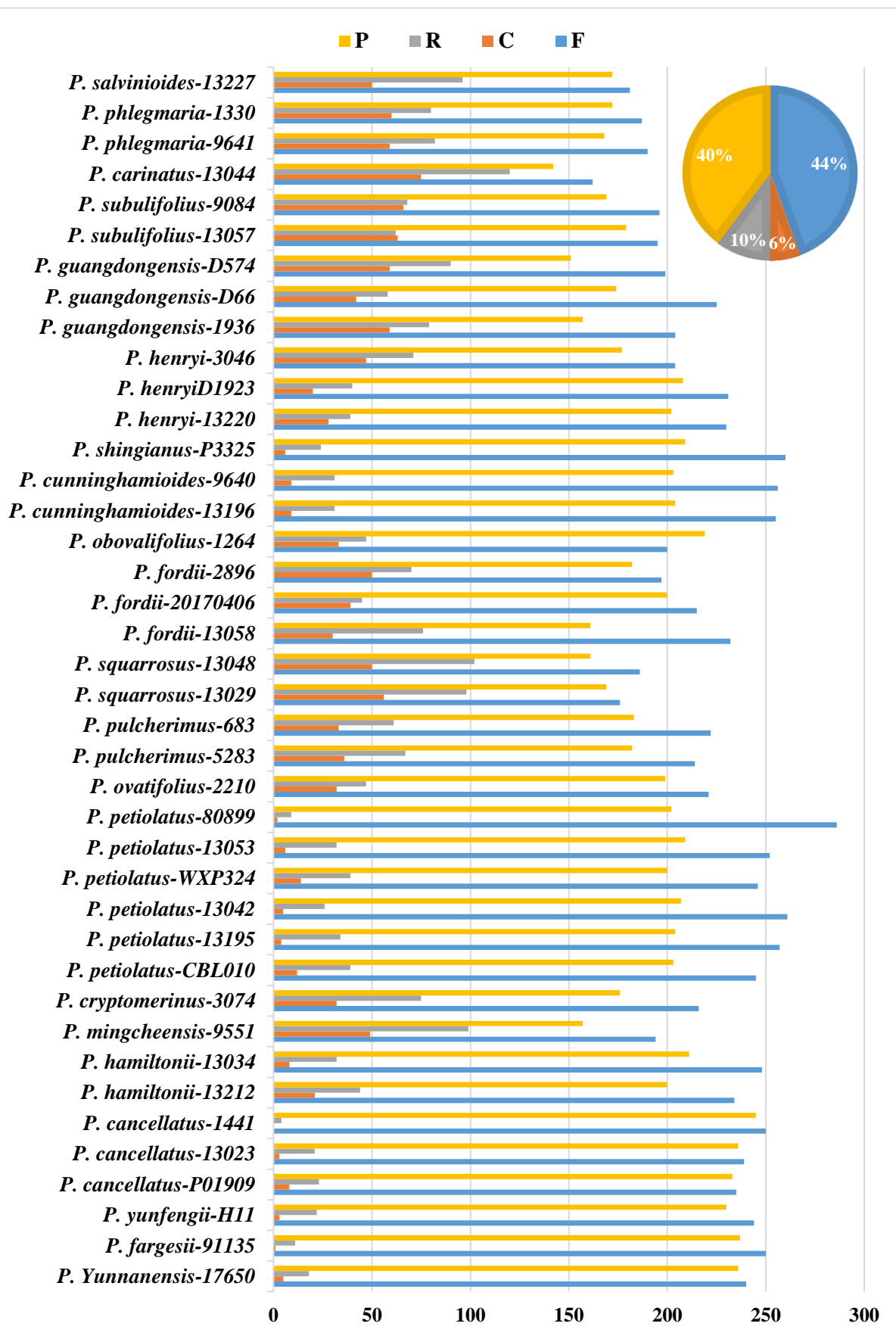

Supplement: Supplementary Figure 4 — Frequency and average proportion of four types of short dispersed repeats (SDRs). Pie chart showing the average proportion of four SDRs types. [file Image4.pdf]

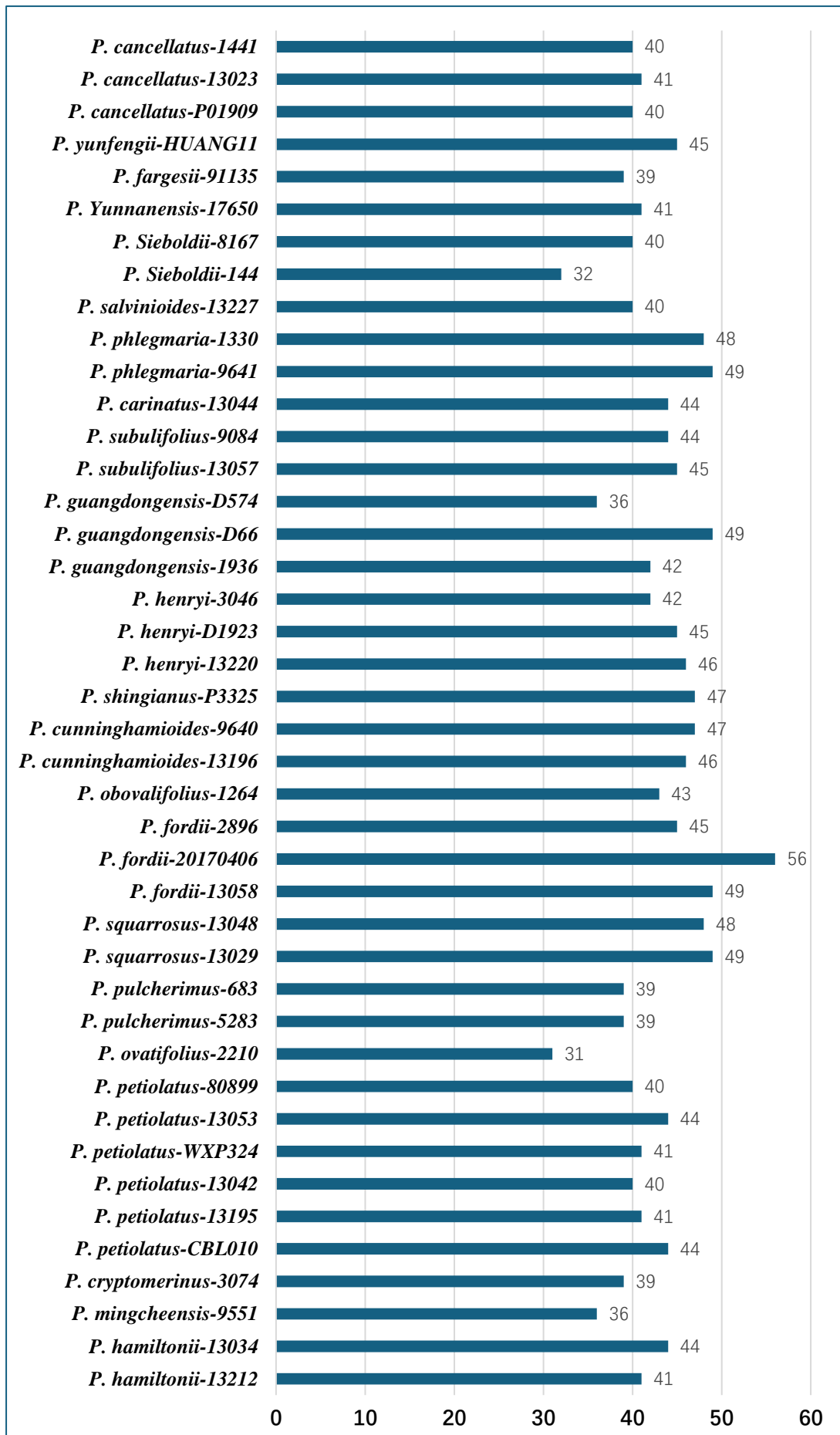

Supplement: Supplementary Figure 5 — Analysis of tandem repeats (TRs) in Phlegmariurus chloroplast genomes. [file Image5.pdf]

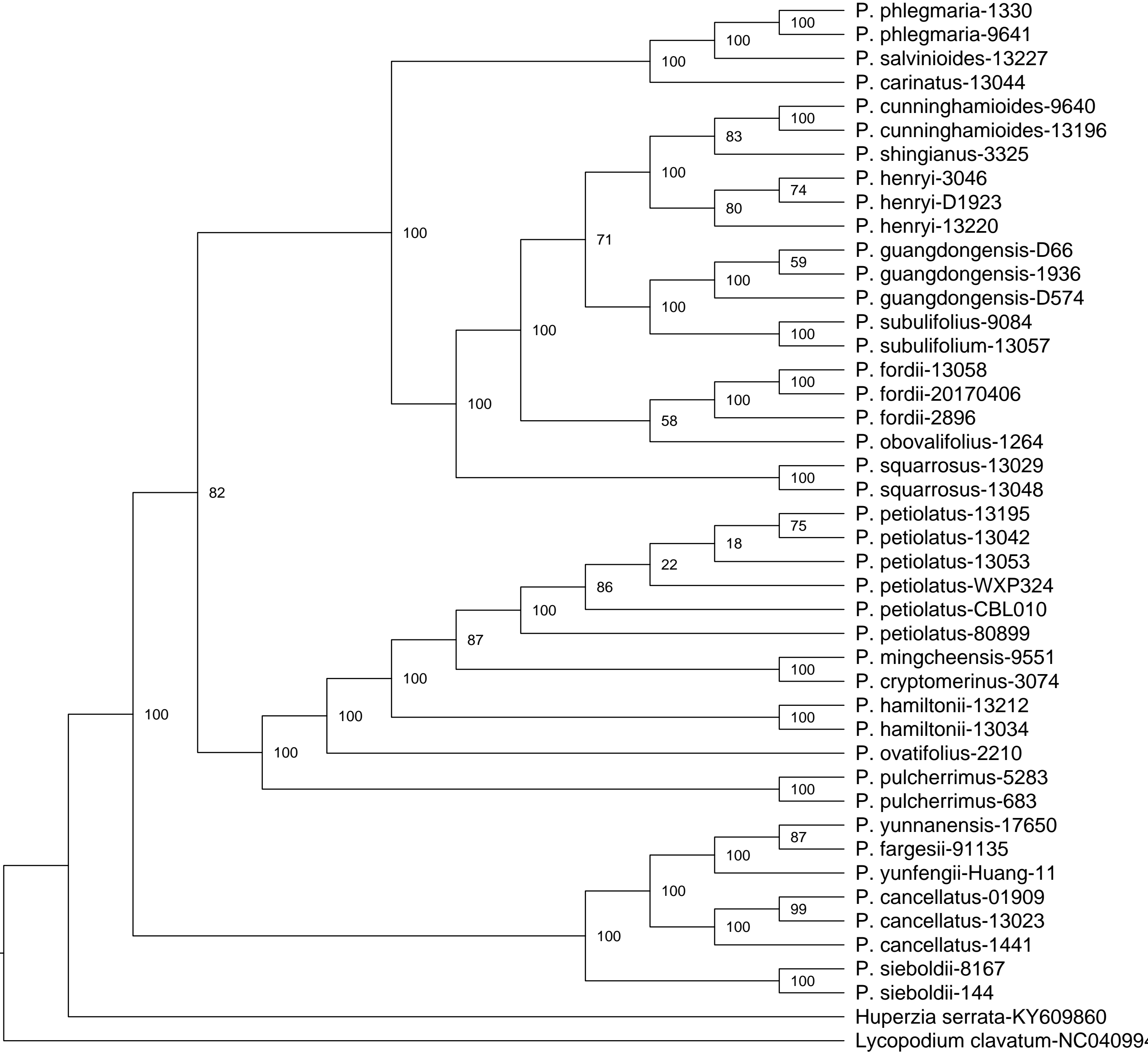

Supplement: Supplementary Figure 6 — Maximum likelihood (ML) cladogram of 42 Phlegmariurus samples inferred from 87 protein-coding genes in chloroplast genome. ML bootstrap (BS) values are shown at each node. [file Image6.pdf]

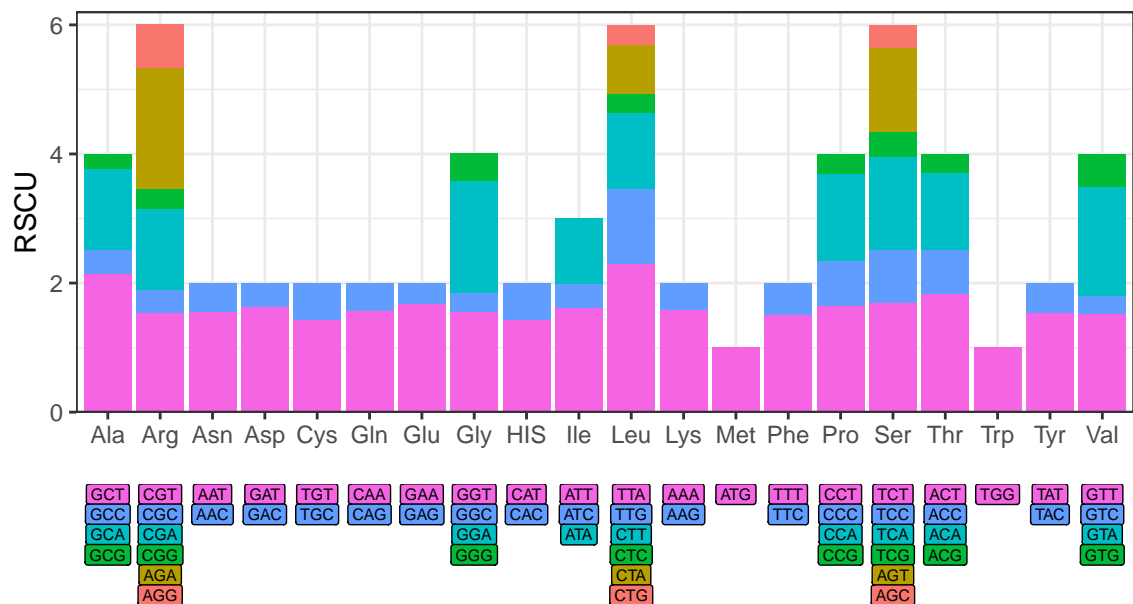

Supplement: Supplementary Figure 7 — The relative synonymous codon usage (RSCU) of P. fargesii calculated based on protein-coding genes. [file Image7.pdf]

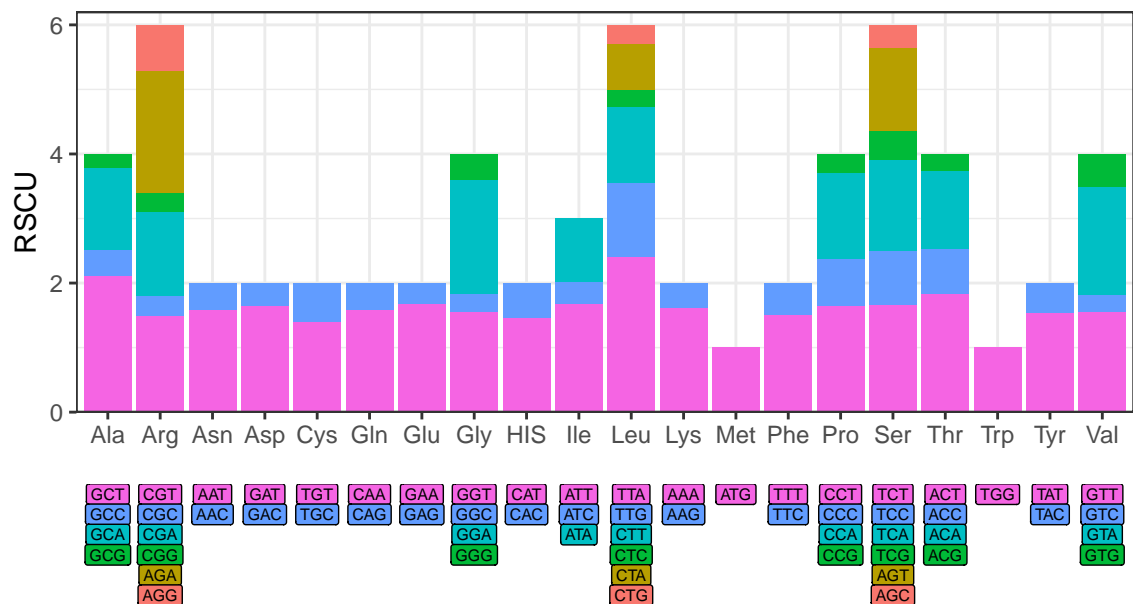

Supplement: Supplementary Figure 8 — The relative synonymous codon usage (RSCU) of P. henryi calculated based on protein-coding genes. [file Image8.pdf]

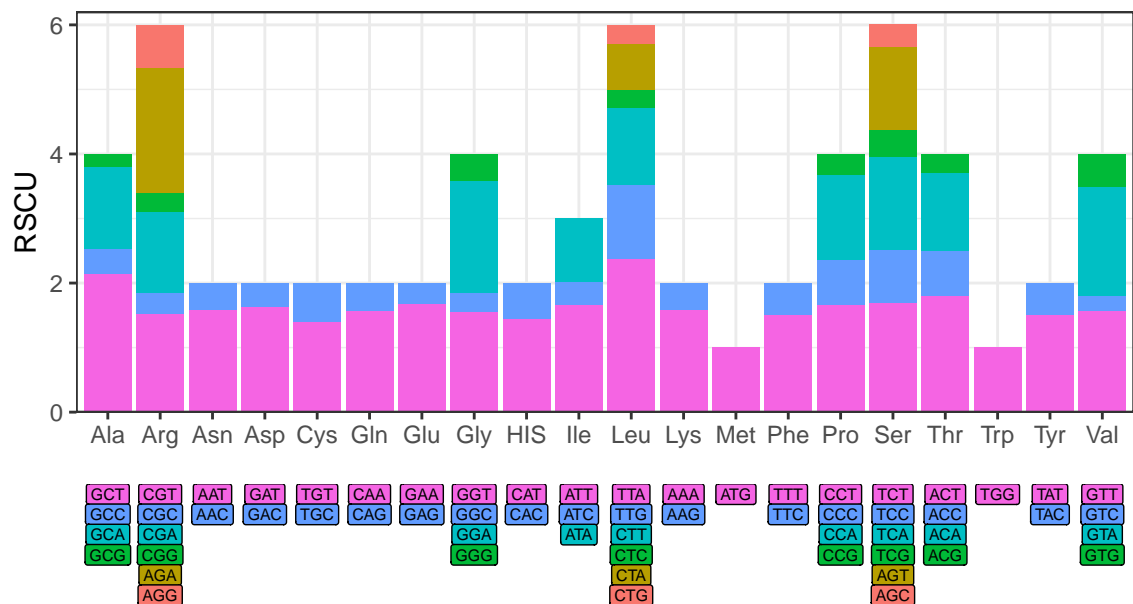

Supplement: Supplementary Figure 9 — The relative synonymous codon usage (RSCU) of P. hamiltonii calculated based on protein-coding genes. [file Image9.pdf]

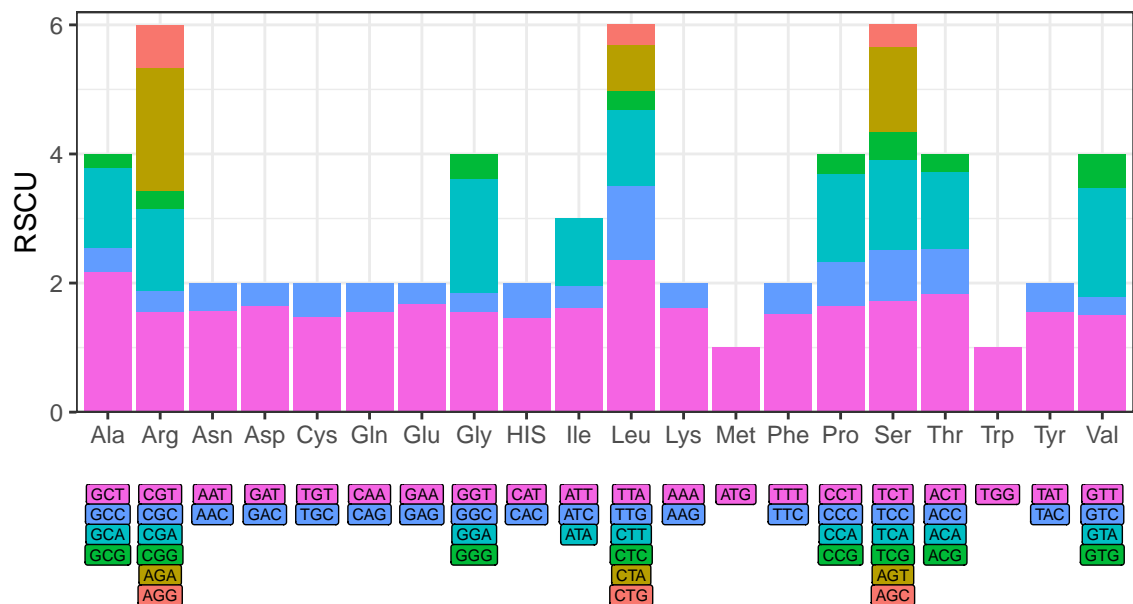

Supplement: Supplementary Figure 10 — The relative synonymous codon usage (RSCU) of P. phlegmaria calculated based on protein-coding genes. [file Image10.pdf]

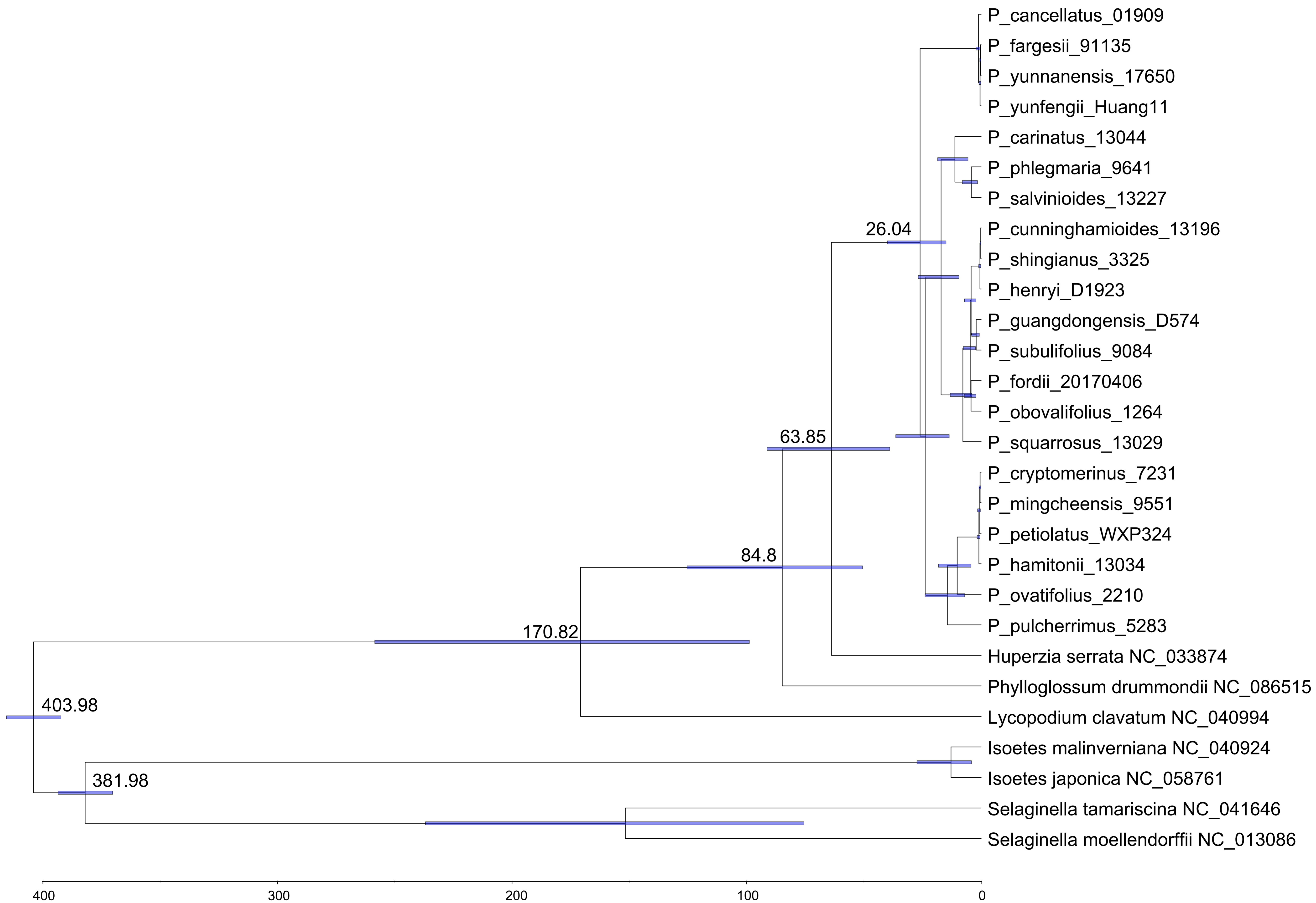

Supplement: Supplementary Figure 11 — Molecular dating of 21 Phlegmariurus species based on the protein-coding sequences in chloroplast genomes. [file Image11.pdf]

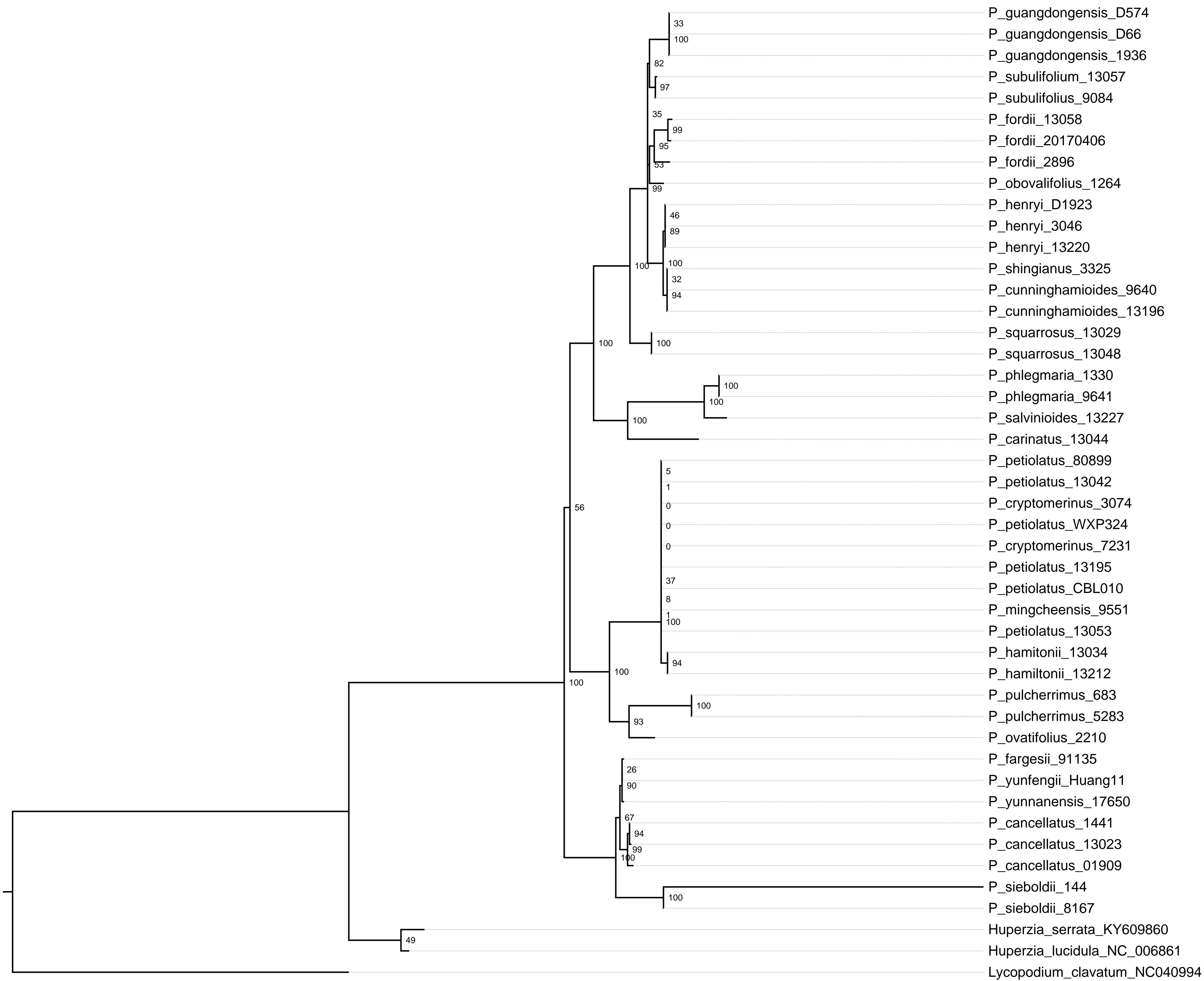

Supplement: Supplementary Figure 12 — Phylogram based on the plastid sequences published in previous studies by Maximum likelihood (ML). Numbers in each nodes represent ML bootstrap values (BS). [file Image12.pdf]
