# Supplementary material for: Comparative chloroplast genomes and phylogenetic analysis of the Phlegmariurus (Lycopodiaceae) from China and neighboring regions
Source: Front Plant Sci. 2025 Jul 8;16:1543431. doi: 10.3389/fpls.2025.1543431 (PMC12279849; doi:10.3389/fpls.2025.1543431)
Supplement: Supplementary file 13 [file DataSheet1.docx]

Table S1 Previous grouping comparisons of *Phlegmariurus* in China.

| Ching, 1982 | | Yang, 1990 | | Zhang & Kung, 1999, 2000 | | Jiang & Zhang, 2023; this study | |
| --- | --- | --- | --- | --- | --- | --- | --- |
| Ser.  *Funiformes* | *P. cancellatus*  *P. cancellatus* var. *minor*  *P. carinatus*  *P. fargesii*  *P. sieboldii*  *P. yunnanensis* | Sect.  *Funiformes* | *P. cancellatus*  *P. carinatus*  *P. fargesii*  *P. sieboldii*  *P. yunnanensis* | Sect.  C*arinaturus* | *P. cancellatus*  *P. carinatus*  *P. fargesii*  *P. sieboldii*  *P. yunnanensis* | Sect.  *Fargesiani* | *P. cancellatus*  *P. fargesii*  *P. sieboldii*  *P. yunfengii*  *P. yunnanensis* |
| Ser.  *Phlegmariae* | *P. guandongensis*  *P. phlegmaria*  *P. salvinioides* | Sect.  *Phlegmariae* | *P. guandongensis*  *P. phlegmaria*  *P. phlegmaria* var. *filiformes*  *P. salvinioides* | Sect.  *Phlegmariurus* | *P. phlegmaria*  *P. salvinioides* | Sect. *Phlegmariurus* | *P. carinatus*  *P. phlegmaria*  *P. salvinioides* |
| Ser. *Hamiltonianae* | *P. fordii*  *P. hamiltonii*  *P. hamiltonii* var. *petiolatus*  *P. henryi*  *P. mingcheensis*  *P. pulcherrimus*  *P. squarrosus*  *P. taiwanensis* | Sect.  *Hamiltonianae* | *P. cryptomerinus*  *P. cunninghamioides*  *P. fordii*  *P. hamiltonii*  *P. henryi*  *P. mingcheensis*  *P. petiolatus*  *P. pulcherrimus*  *P. qiongzhongensis*  *P. shangsiensis*  *P. squarrosus*  *P. taiwanensis* | Sect.  *Huperzioides* | *P. austrosinicus*  *P. cryptomerinus*  *P. cunninghamioides*  *P. fordii*  *P. hamiltonii*  *P. henryi*  *P. mingcheensis*  *P. ovatifolius*  *P. petiolatus*  *P. pulcherrimus*  *P. shangsiensis*  *P. squarrosus*  *P. taiwanensis* | Sect. *Hamiltoniani* | *P. cryptomerinus*  *P. hamiltonii*  *P. mingcheensis*  *P. ovatifolius*  *P. petiolatus*  *P. pulcherrimus* |
|  |  |  |  |  |  | Sect. *Squarrosurus* | *P. fordii*  *P. guandongensis*  *P. henryi*  *P. squarrosus*  *P. subulifolius*  *P. singianus*  *P. cunninghamioides*  *P. obovalifolius* |
