# Supplementary material for: Comparative chloroplast genomes and phylogenetic analysis of the Phlegmariurus (Lycopodiaceae) from China and neighboring regions
Source: Front Plant Sci. 2025 Jul 8;16:1543431. doi: 10.3389/fpls.2025.1543431 (PMC12279849; doi:10.3389/fpls.2025.1543431)
Supplement: Supplementary file 19 [file DataSheet7.docx]

**Table S7** **Likelihood ratio test (LRT) of the variable ω ratio under different models.**

| gene | comparisons | 2Δl | df | p |
| --- | --- | --- | --- | --- |
| *atpB** | M1 vs M2 | 6.67696 | 2 | 3.54908e-02 |
|  | M7 vs M8 | 7.02892 | 2 | 2.97638e-02 |
| *cemA*** | M1 vs M2 | 9.63624 | 2 | 8.08199e-03 |
|  | M7 vs M8 | 9.60730 | 2 | 8.19978e-03 |
| *chlB*** | M1 vs M2 | 37.10484 | 2 | 8.76566e-09 |
|  | M7 vs M8 | 37.17064 | 2 | 8.48200e-09 |
| *chlL** | M1 vs M2 | 6.66568 | 2 | 3.56916e-02 |
|  | M7 vs M8 | 6.97268 | 2 | 3.06128e-02 |
| *chlN*** | M1 vs M2 | 27.41330 | 2 | 1.11501e-06 |
|  | M7 vs M8 | 29.32554 | 2 | 4.28585e-07 |
| *ndhB*** | M1 vs M2 | 29.4007 | 2 | 4.12781e-07 |
|  | M7 vs M8 | 29.41024 | 2 | 4.10814e-07 |
| *petL*** | M1 vs M2 | 11.42236 | 2 | 3.30876e-03 |
|  | M7 vs M8 | 11.14032 | 2 | 3.80984e-03 |
| *psbC** | M1 vs M2 | 7.79436 | 2 | 2.02990e-02 |
|  | M7 vs M8 | 8.98368 | 2 | 1.12001e-02 |
| *psbM** | M1 vs M2 | 6.53752 | 2 | 3.80533e-02 |
|  | M7 vs M8 | 6.62390 | 2 | 3.64448e-02 |
| *rbcL** | M1 vs M2 | 7.86408 | 2 | 1.96035e-02 |
|  | M7 vs M8 | 8.52050 | 2 | 1.41188e-02 |
| *rpoB** | M1 vs M2 | 6.10580 | 2 | 4.72216e-02 |
|  | M7 vs M8 | 6.64100 | 2 | 3.61345e-02 |
| *ycf1*** | M1 vs M2 | 76.36016 | 2 | 4.00000e-17 |
|  | M7 vs M8 | 76.36392 | 2 | 4.00000e-17 |
